# Seasonal soil microbial responses are limited to changes in functionality at two Alpine forest sites differing in altitude and vegetation

**DOI:** 10.1038/s41598-017-02363-2

**Published:** 2017-05-19

**Authors:** José A. Siles, Rosa Margesin

**Affiliations:** Institute of Microbiology, University of Innsbruck, Technikerstrasse 25, A-6020 Innsbruck, Austria

## Abstract

The study of soil microbial responses to environmental changes is useful to improve simulation models and mitigation strategies for climate change. We here investigated two Alpine forest sites (deciduous forest *vs*. coniferous forest) situated at different altitudes (altitudinal effect) in spring and autumn (seasonal effect) regarding: (i) bacterial and fungal abundances (qPCR); (ii) diversity and structure of bacterial and fungal communities (amplicon sequencing); and (iii) diversity and composition of microbial functional gene community (Geochip 5.0). Significant altitudinal changes were detected in microbial abundances as well as in diversity and composition of taxonomic and functional communities as a consequence of the differences in pH, soil organic matter (SOM) and nutrient contents and soil temperatures measured between both sites. A network analysis revealed that deciduous forest site (at lower altitude) presented a lower resistance to environmental changes than that of coniferous forest site (at higher altitude). Significant seasonal effects were detected only for the diversity (higher values in autumn) and composition of microbial functional gene community, which was related to the non-significant increased SOM and nutrient contents detected in autumn respect to spring and the presumable high capacity of soil microbial communities to respond in functional terms to discreet environmental changes.

## Introduction

Forests, which cover 38 million square kilometers of the planet and contain more than three trillions of trees, have a crucial role on the climate, the geochemical cycles and especially on the global carbon (C) cycling since they act as important C sinks^1^. For example, C fixed by primary producers in forests exceeds C loss by respiration by 7–25%^2^ and the current C stocks in the world’s forests have been estimated to be 861 ± 66 Pg C^3^. Nonetheless, Earth’s climate is warming and this increase in global temperature will probably have consequences on the balance between annual soil C inputs (photosynthesis) and losses (respiration)^4^. In this balance, it is especially relevant the role of soil microorganisms, key players in soil organic matter (SOM, the main C reservoir in forests) transformation and decomposition, and, as a consequence, in soil respiration^5, 6^. Despite their pivotal role, our knowledge regarding how soil microorganisms respond to environmental changes is still scarce^7^, and microbial dynamics have only recently started to be incorporated in ecosystem models^8^. As the predictive capacity of soil carbon cycling models enhances when microbial ecology aspects are considered, it is evident that soil microorganisms are essential for understanding and predicting ecosystem processes^9^.

Mountain ecosystems are characterized by drastic changes in climate and biotic characteristics over short altitudinal and geographical distances, and have thus been regarded as powerful natural experiments to study soil microbial response to environmental changes^10, 11^. In the last few years, with the rapid development of high-throughput sequencing techniques, many studies have documented soil bacterial^12–14^, archaeal^15, 16^ and/or fungal^17–19^ altitudinal patterns as well as the factors driving these patterns over a high variety of mountain ecosystems. Most of these studies are mainly focused on taxonomic traits of microbial communities; however, if functional traits of microbial communities are also assessed, the useful information that can be extracted from them for climate change mitigation models will increase^20^. Patterns of microbial functional signatures, such as structural genes relevant to metabolic pathways, energetics and regulatory circuits, provide a better understanding of ecosystem functions, biogeochemical cycles and microbial responses to environmental changes^21, 22^. In this context, GeoChip, a microarray-based metagenomic technology, has shown to be a specific and sensitive tool for studying microbial gene diversity and metabolic potential in many different environments^23, 24^. In fact, previous surveys have already used this technology to investigate the effect of altitude on microbial functional gene community^21^. GeoChip has also been applied along with gene amplicon sequencing to assess both functional and taxonomic properties of soil microbial communities at different altitudes. Ding, *et al*.^25^ found significant differences in soil bacterial (based on Illumina amplicon sequencing of 16S rRNA genes) and microbial functional (GeoChip-based) community composition and diversity between shrubland and coniferous forests at the forest timberline. While, Shen, *et al*.^22^, studying an altitudinal gradient on Changbai Mountain in China, discovered that microbial functional gene richness exhibited a dramatic increase at the treeline ecotone, but the 16S-rRNA gene sequencing-based bacterial taxonomic diversity did not show a similar pattern.

Seasonal aspects of soil microbial communities are generally neglected in works correlating altitude with taxonomic and functional patterns of microbial communities. Nonetheless, seasonality is related to alternating climatic conditions and shapes belowground C, nitrogen (N) and other nutrients transfer by changes in tree physiology. Trees release large proportions of their accumulated C and N in the form of tree residues (e.g., litter and dead roots) and root exudates to the soil^26^. The importance of season is also dependent on the type of vegetation, whose composition varies with altitude^10^. Deciduous forests are characterized by the photosynthetic activity of trees only during the vegetative period and a short litterfall period in autumn. In contrast, coniferous forests present a longer vegetative period and enrich the soil with a more recalcitrant litter compared to that of deciduous vegetation^27, 28^. Therefore, integrative surveys, where taxonomic and functional traits of soil bacterial and fungal communities are studied in response to changing environmental conditions (such as those related to shifts in altitude) and taking into consideration seasonal aspects, can contribute useful and reliable information for improvement of simulation models and mitigation strategies for climate change.

Our recent investigations have been focused on the characterization of altitudinal and/or seasonal (spring to autumn comparisons) shifts in soil microbial communities using different methodological approaches at four Alpine forest sites (representing a climosequence) located over an altitudinal gradient from 545 to 2,000 m above sea level (asl). Using this altitudinal gradient: (i) Siles & Margesin^17^ confirmed the significance of altitude determining changes in abundance and Illumina-based analysis of diversity and composition of bacterial and fungal communities (with no significant changes in the case of archaeal communities) and elucidated the main driving factors of those changes considering only one season; (ii) França, *et al*.^29^ reported for the first time culturable bacterial and yeast diversity of Alpine forest soils and discovered distinct differences between culturable bacterial and yeast diversity related to altitude, season and isolation temperature; and (iii) Siles, *et al*.^30^ described significant altitudinal and seasonal shifts in some soil physicochemical properties and PLFA (phospholipid fatty acid)-based abundance and structure of soil microbial communities as well as altitudinal variations (with limited seasonal effects) in soil microbial functionality (determined through measurement of potential enzyme activities and characterization of community level physiological profiles). Notwithstanding all these works, there is still a lack of detailed information regarding the altitudinal and seasonal shifts in taxonomic diversity and composition of both bacterial and fungal communities as well as their functional traits in the aforementioned forest ecosystems using comprehensive and state-of-the-art tools.

We here studied two Alpine forests situated at two different altitudes and differing in the vegetation cover type (deciduous forest *vs*. coniferous forest) in spring and autumn to evaluate the effect of altitude and/or season on: (i) soil physicochemical properties and soil temperature; (ii) bacterial and fungal abundance (through qPCR); (iii) diversity and structure of bacterial and fungal communities (using Illumina amplicon sequencing); (iv) network structures of bacterial and fungal communities (using a random matrix theory-based approach); and (iv) microbial diversity and structure of potential functional gene community (using GeoChip 5.0). Finally, we aimed to discover the main factors explaining the altitudinal and seasonal shifts in abundance, diversity and composition of bacterial and fungal communities as well as in microbial functional gene community. We hypothesized that the significantly different bacterial and fungal communities characterizing these two forest sites would differently respond in taxonomic and functional terms to the changes in soil physicochemical properties and environmental conditions determined by seasonality.

## Results

### Soil physicochemical and temperature properties

Deciduous forest site M and coniferous forest site R significantly differed in all the physicochemical properties tested, with the exception of the content in Mg (Table 1). Both sites presented an acidic pH, although that of subalpine site R was significantly lower. Increased levels of EC (electrical conductivity), SOM and other nutrients (including inorganic forms of N) as well as a higher C/N ratio were detected at site R. Regarding the effect of season, there was a trend of increased SOM and nutrient contents in autumn compared to spring at both sites; however, these increments were not significant except in the case of NO_3_^−^ -N (Table 1).

**Table 1:** Soil physicochemical properties, bacterial and fungal abundances and soil temperature measurements determined at the deciduous forest site M (545–570 m asl) and the coniferous forest site R (1,724–1,737 m) in spring and autumn.

	Site M		Site R		p-value
	Spring	Autumn	Spring	Autumn	
Soil physicochemical properties					
pH	4.75 b	4.91 b	3.34 a	3.17 a	**<0.0001**
EC^1^ (mg KCl kg^−1^ dw soil)	186 a	170 a	381 b	469 b	**<0.0001**
SOM^2^ (%)	12.35 a	15.15 a	32.15 b	37.95 b	**<0.0001**
TOC^3^ (%)	7.18 a	8.79 a	18.70 b	22.05 b	**<0.0001**
N (%)	0.41 a	0.45 a	0.90 b	1.10 b	**<0.0001**
NH_4_^+^-N (mg kg^−1^ dw soil)	16.38 a	20.07 ab	26.26 b	28.79 b	**<0.0001**
NO_3_^−^-N (mg kg^−1^ dw soil)	8.62 a	17.86 b	27.76 b	43.09 c	**<0.0001**
C/N	18.91 a	20.03 ab	22.18 ab	22.40 b	**0.0233**
P (mg kg^−1^ dw soil)	17.00 a	21.50 a	22.50 ab	36.00 b	**0.0025**
K (mg kg^−1^ dw soil)	90 a	100 a	210 b	252 b	**<0.0001**
Mg (mg kg^−1^ dw soil)	196 a	213 a	200 a	174 a	0.8245
Microbial abundance					
Bacterial community (16S rRNA gene copy number g^−1^ dw soil)	2.19 × 10^11^ a	2.43 × 10^11^ a	2.56 × 10^11^ b	2.73 × 10^11^ b	**<0.0001**
Fungal community (18S rRNA gene copy number g^−1^ dw soil)	3.25 × 10^8^ a	3.53 × 10^8^ a	5.86 × 10^8^ b	6.94 × 10^8^ b	**<0.0001**
**Soil temperature**	**Site M**		**Site R**		**p-value**
Mean soil temperature (°C)	12.2 b		6.3 a		**<0.0001**
Maximum soil temperature (°C)	16.2 b		12.1 a		**0.0001**
Minimum soil temperature (°C)	8.9 b		3.0 a		**<0.0001**

For ANOVA analyses, p-values in bold denote statistical significance (p ≤ 0.05); for Tukey’s HSD tests, values followed by different letters are significantly different (p ≤ 0.05).

^1^EC, electrical conductivity.

^2^SOM, soil organic matter.

^3^TOC, total organic carbon.

Monitoring of soil temperature of these two sites demonstrated that mean, maximum and minimum soil temperatures of subalpine site R were significantly lower than those of submontane site M (Table 1).

### Bacterial and fungal abundances

The abundance of bacterial and fungal communities was significantly higher at coniferous forest site R compared to deciduous forest site M, and although the 16S rRNA and 18 rRNA gene copy numbers increased in autumn respect to spring at both sites, these variations were not significant (Table 1).

### Taxonomic characteristics of bacterial and fungal communities

**Figure 1:**
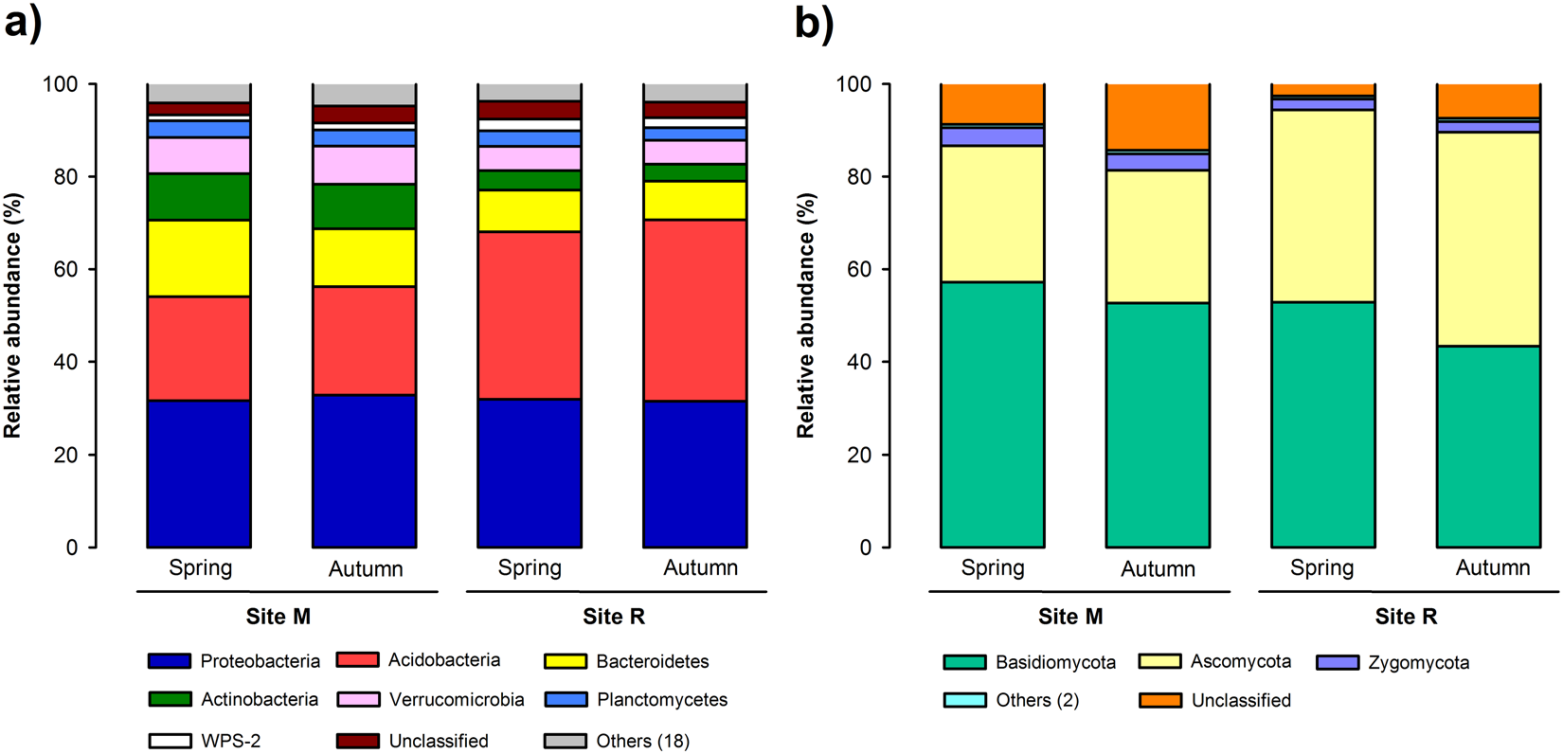
Relative abundance of the bacterial (**a**) and fungal (**b**) phyla found at the deciduous forest site M (545–570 m asl) and the coniferous forest site R (1,724–1,737 m) in spring and autumn.

Across the 24 soil samples analyzed, we obtained a total of 871,530 high-quality bacterial sequences distributed among 4603 OTUs (operational taxonomic unit), with an average number of sequences per sample of 36,314 (standard deviation, SD = 6493). Total bacterial diversity was distributed among 25 different phyla, although the five most dominant phyla, *Proteobacteria* (the number of classified sequences in this phylum ranged from 23.81 to 40.01% across the 24 samples), *Acidobacteria* (9.43–44.32%), *Bacteroidetes* (4.99–27.79%), *Actinobacteria* (2.99–14.34%) and *Verrucomicrobia* (4.00–10.96%), accounted for more than 82% of the total number of sequences in all the samples (Fig. 1a). *Alphaproteobacteria, Gammaproteobacteria* and *Betaproteobacteria* classes dominated among *Proteobacteria*; Gp1, Gp2 and Gp3 subgroups among *Acidobacteria*; *Sphingobacteriia* class among *Bacteroidetes*; and *Actinobacteridae* and *Rubrobacteridae* subclasses among *Actinobacteria* (Fig. S1a).

Regarding the fungal community, Illumina amplicon sequencing analysis yielded 2,012,629 high-quality sequences across the 24 samples, which were distributed among 3,400 different OTUs. The average number of sequences per sample was 83,860 (SD = 31,431). Fungal diversity was distributed among 5 different phyla, and the predominant ones were *Basidiomycota* (the number of classified sequences in this phylum ranged from 14.46 to 88.07% across the 24 samples), *Ascomycota* (5.28–88.07%) and *Zygomycota* (0.62–8.42%), which accounted for more than 80% of total sequences (Fig. 1b). The predominant classes were *Agaricomycetes* among *Basidiomycota*; *Eurotiomycetes*, *Leotiomycetes* and *Pezizomycetes* among *Ascomycota*; and Incertae sedis 10 (which includes *Mortierellales* and *Mucorales* orders) among *Zygomycota* (Fig. S1b).

### Effect of altitude and season on diversity and structure of bacterial communities

Bacterial richness, Shannon index and the richness estimators Chao1 and ACE were significantly higher at submontane site M compared to subalpine site R (Table 2). However, bacterial evenness was significantly higher at site R. No significant seasonal changes were detected for any of the parameters calculated at any of the sites.

**Table 2:** Diversity characteristics of bacterial, fungal and microbial functional gene communities determined at the deciduous forest site M (545–570 m asl) and the coniferous forest site R (1,724–1,737 m) in spring and autumn.

	Site M		Site R		p-value
	Spring	Autumn	Spring	Autumn	
Bacterial community					
Mean sequence number	39831	32096	41064	32264	
Richness	1874 b	1977 b	1332 a	1244 a	**<0.0001**
Shannon index	5.91 b	6.07 b	5.34 a	5.28 a	**<0.0001**
Evenness	0.398 a	0.388 a	0.438 b	0.446 b	**<0.0001**
Chao 1	2577 b	2603 b	1917 a	1795 a	**<0.0001**
ACE	2587 b	2629 b	2131 a	2001 a	**0.0002**
Fungal community					
Mean sequence number	59830	62870	97895	114843	
Richness	998 a	1141 a	978 a	1004 a	0.1240
Shannon index	3.67 ab	4.23 b	3.30 a	3.43 a	**0.0009**
Evenness	0.445 a	0.447 a	0.431 a	0.432 a	0.0622
Chao 1	1490 a	1568 a	1433 a	1469 a	0.2866
ACE	1661 a	1619 a	1497 a	1505 a	0.2344
Microbial functional gene community					
Richness	14547 a	15046 b	15237 c	15560 d	**<0.0001**
Sahnnon index	8.21 a	8.42 b	8.63 c	8.93 d	**<0.0001**
Evenness	0.937 a	0.936 a	0.937 a	0.937 a	0.821

For ANOVA analyses, p-values in bold denote statistical significance (p ≤ 0.05); for Tukey’s HSD tests, mean values followed by different letters are significantly different (p ≤ 0.05).

**Figure 2:**
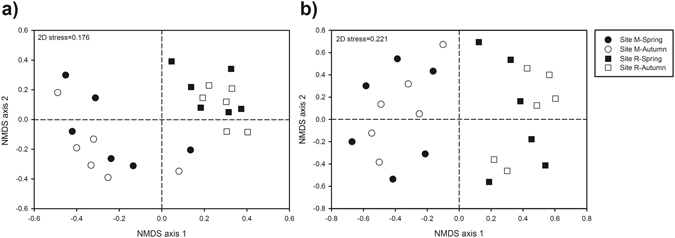
Nonmetric multidimensional scaling (NMDS) ordinations based on Bray-Curtis similarities of OTU-based bacterial (**a**) and fungal (**b**) community structures found at the deciduous forest site M (545–570 m asl) and the coniferous forest site R (1,724–1,737 m) in spring and autumn.

PERMANOVA and ANOSIM analyses demonstrated that bacterial community structure was significantly affected by altitude (PERMANOVA, F = 21.21, *p* = 0.0001; ANOSIM, R = 0.89, *p* = 0.0001), but not by season (PERMANOVA, F = 0.70, *p* = 0.5402; ANOSIM, R = −0.02, *p* = 0.5984), nor by the interaction altitude × season (PERMANOVA, F = 0.77, *p* = 0.4619). These results were corroborated by NMDS ordination; NMDS axis 1 clearly separated samples from deciduous forest site M in spring and autumn from those of coniferous forest site R also in both seasons, evidencing no effect of season on bacterial community structure (Fig. 2a). The differences in bacterial community composition between both sites were also evidenced at taxonomic level. Among the seven most abundant bacterial phyla, *Bacteroidetes* and *Actinobacteria* were more abundant at submontane site M respect to subalpine site R, while the opposite was noted for *Acidobacteria* (Table S1). At class level, *Alpha-* and *Betaproteobacteria*, *Actinobacteria* (*Actinobacteridae* subclass) and *Spartobacteria* as well as subgroup Gp6 of *Acidobacteria* were present at a higher relative abundance at deciduous forest site M; whereas, acidobacterial subgroups Gp1 and Gp2, *Gammaproteobacteria* and subdivision 3 of *Verrucomicrobia* were detected to a significantly higher extent at coniferous forest site R. Significantly seasonal variations were not noticed for any of the aforementioned taxonomic groups at any of the sites (Table S1). The detailed taxonomic analysis of the top 76 most abundant bacterial OTUs (i.e., those OTUs with an abundance ≥0.25% across the 24 samples considering the total number of reads), allowed the assignment of 42 of them at genus level (Table S2). OTUs belonging to genera such as *Bradyrhizobium*, *Rhodoplanes*, *Variibacter* and *Nitrospirillum* (*Alphaproteobacteria*); *Mycobacterium* and *Asanoa* (*Actinobacteria*); as well as *Tepidimonas* (*Betaproteobacteria*) were more abundant at submontane site M in comparison with subalpine site R. Instead, OTUs classified as *Povalibacter* and *Permianibacter* (*Gammaproteobacteria*); *Rhodoplanes* and *Roseiarcus* (*Alphaproteobacteria*); *Telmatobacter*, *Acidipila* and candidatus *Solibacter* (*Acidobacteria*); *Limisphaera* (*Verrucomicrobia*); as well as *Labilithrix* (*Deltaproteobacteria*) were present to a higher extent at subalpine site R. Only 2 of the top 76 most abundant OTUs showed significant seasonal effects; OTU10 (belonging to subgroup Gp2 of *Acidobacteria*) and OTU41 (classified as *Steroidobacter* (*Gammaproteobacteria*)) were more abundant in autumn than in spring at subalpine site R (Table S2).

### Effect of altitude and season on diversity and structure of fungal communities

Fungal richness, evenness and richness estimators Chao1 and ACE did not show significant altitudinal of seasonal variations. Only a significantly higher Shannon index was detected at deciduous forest site M in autumn (Table 2).

PERMANOVA and ANOSIM analyses demonstrated that OTU-based fungal community structure significantly varied by altitude (PERMANOVA, F = 17.37, *p* = 0.0001; ANOSIM, R = 0.33, *p* = 0.0001), but not by season (PERMANOVA, F = 0.89, *p* = 0.5713; ANOSIM, R = −0.03, *p* = 0.6403), nor by the interaction altitude × season (PERMANOVA, F = 0.81, *p* = 0.7116). In concordance with these results, fungal NMDS grouped the samples in two different clusters, one of them comprising the samples of deciduous forest site M in spring and autumn and another one including the samples from coniferous forest site R in both seasons (Fig. 2b). These results were also evidenced at taxonomic level. *Thelephorales*, *Pezizales*, *Mucorales*, *Hysteriales* and *Geminibasidiales* orders were significantly more abundant at submontane site M, while *Eurotiales* predominated at subalpine site R (Table S3). Significant seasonal changes were not noticed for any of these taxonomic groups. The taxonomic assignment of the top 91 most abundant fungal OTUs (i.e., those OTUs with an abundance ≥0.25% across the 24 samples considering the total number of reads) allowed the classification of 64 of them at genus level (Table S4). OTUs belonging to *Amanita* (OTU6), *Geminibasidium* (OTU58), *Umbelopsis* (OTU46) and *Pachyphloeus* (OTU54) genera were more abundant at deciduous forest site M, while OTUs classified at genus level as *Elaphomyces* (OTU1), *Russula* (OTU4) or *Mortierella* (OTU61) were found to a higher extent at coniferous forest site R (Table S4). No significant seasonal effects were found for any of the top 91 most abundant fungal OTUs at any of the sites.

### Network analysis of bacterial and fungal communities

**Figure 3:**
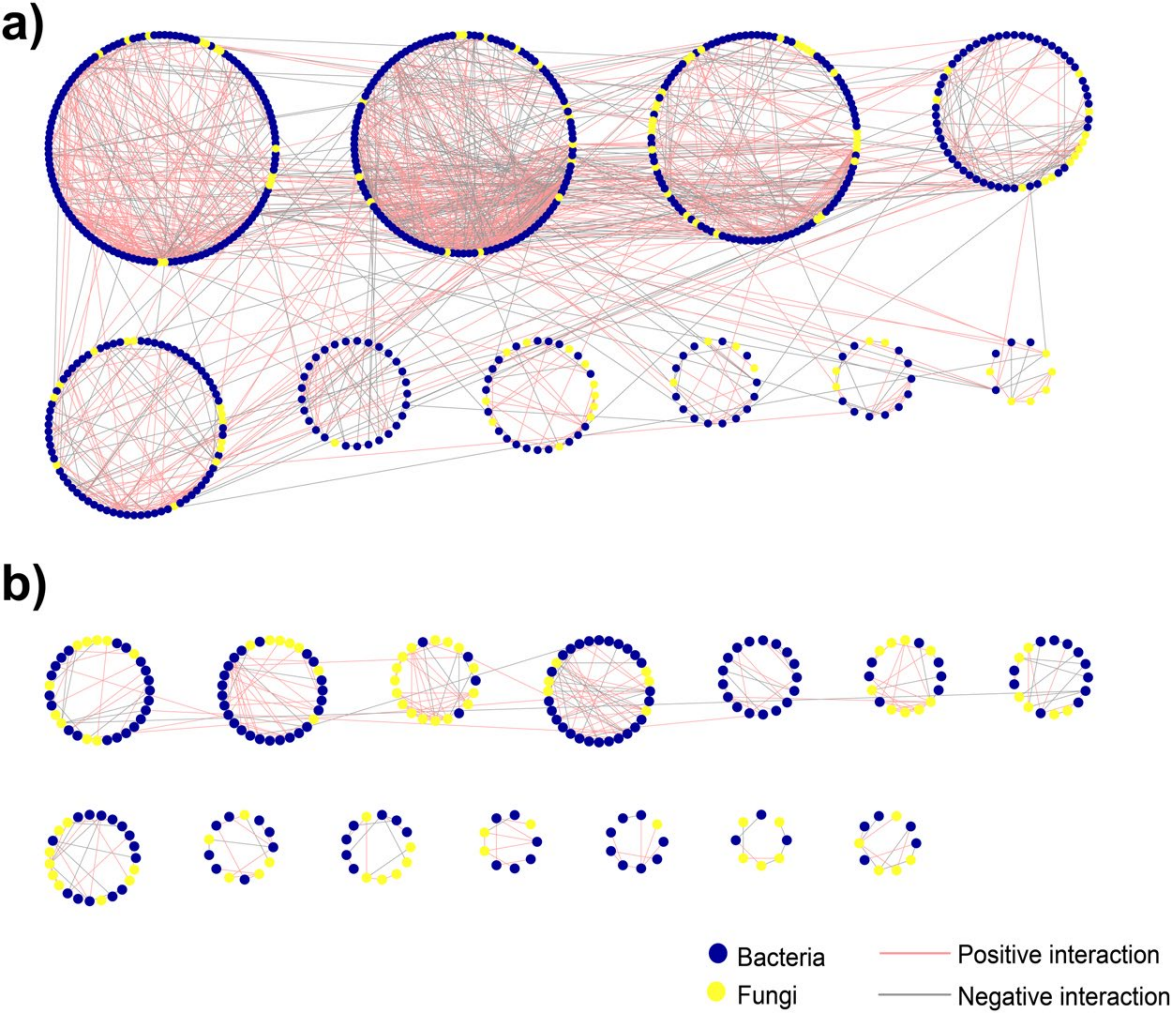
Network interaction graphs for the deciduous forest site M (545–570 m asl) (**a**) and the coniferous forest site R (1,724–1,737 m) (**b**) based on random matrix theory analyses from OTU-based bacterial and fungal community structures. Each node represents a bacterial (blue) or fungal (yellow) OTU. A line connecting two nodes prepresents the positive (red) or negative (gray) interaction between them. OTUs were separated into different modules, shown as circles, by the greedy modularity optimization method. Only modules ≥10 nodes have been represented.

Network analysis for each site, including data of both bacterial and fungal community structures in both season (since a significant seasonal effect was not detected) (Fig. 3), revealed that the number of nodes and edges of the deciduous forest site M network (Fig. 3a; Table 3) was much higher than that of the coniferous forest site R network (Fig. 3b; Table 3), although the nodes classified as Bacteria dominated at both sites (Fig. 3). Both networks fitted well with the power-law model, indicating their scale-free properties (Table 3). The average clustering coefficient (avgCC) and average path distance (GD) of both site networks were significantly different from corresponding randomized networks, which would be indicating their small-world behavior^31^. The average degree (avgK) in the network of submontane site M, a key topological property to describe how well a node is connected with the others, was higher (ca. twofold) than that of the subalpine site R, suggesting the existence of a more complex microbial network and coupling at submontane site M. On the contrary, an increased modularity value, as a measurement of system resistance, was noticed at coniferous forest site R (Table 3).

**Table 3:** Major network characteristics of bacterial and fungal communities found at the deciduous forest site M (545–570 m asl) and the coniferous forest site R (1,724–1,737 m).

	Network properties	Site M	Site R
Empirical networks	Original number of bacterial and fungal OTUs	6880	5502
	Similarity threshold	0.900	0.900
	Total nodes	826	468
	Total links	1864	461
	Links per node	2.256	0.985
	*R* ^2^ of power law	0.923	0.937
	Average degree (avgK)	4.513	1.971
	Average clustering coefficient (avgCC)	0.162	0.097
	Average path distance (GD)	5.364	7.571
	Modularity (M)	0.675	0.925
Random networks	Average clustering coefficient (avgCC)	0.014 ± 0.002	0.003 ± 0.002
	Average path distance (GD)	4.119 ± 0.030	6.990 ± 0.298
	Modularity (M)	0.465 ± 0.004	0.836 ± 0.008

The Zi-Pi plot illustrating the topological roles of individual network nodes showed that the majority of nodes from both networks were categorized as peripherals (specialists), with only a few links and almost always linked to the nodes within their own modules (i.e., Pi = 0) (Fig. S2). A total of 19 module hubs and 20 connectors (i.e., key nodes in structuring networks) were found at deciduous forest site M (Fig. S2a). These 39 generalist nodes were composed of 6 *Acidobacteria* (subgroups Gp1, Gp2, Gp3 and Gp16), 3 *Actinobacteria*, 5 *Alphaproteobacteria*, 3 *Betaproteobacteria*, 2 *Deltaproteobacteria*, 3 *Gammaproteobacteria*, 1 *Planctomycetia*, 7 *Sphingobacteriia*, 1 WPS-2 and 2 unclassified OTUs among Bacteria, as well as 1 *Leotiomycetes*, 2 *Pezizomycetes*, 1 *Sordariomycetes* and 2 unclassified OTUs, among Fungi (Table S5). At the coniferous forest site R, 6 module hubs and 1 connector were detected (Fig. S2b). They were taxonomically identified as *Gammaproteobacteria* (1) and *Actinobacteria* (1) among Bacteria, as well as *Agaricomycetes* (1) and *Eurotiomycetes* (1) among Fungi; 1 bacterial node and 2 fungal nodes were unclassified OTUs (Table S5).

### **Effect of altitude and season on diversity and structure of microbial functional gene communities**

A total of 20,729 functional genes were detected by GeoChip 5.0 across the 12 samples analyzed, with an average number of detected genes per sample of 15,098 (SD = 385). These genes were mainly involved in six functional categories: C cycling (41.88%), organic remediation (23.26%), N cycling (12.91%), sulfur (S) cycling (8.03%), metal homeostasis (7.32%) and phosphorus (P) cycling (4.42%) (Table S6). Other three different categories (virulence, other functions and secondary metabolism) were also detected but in a very low proportion.

**Figure 4:**
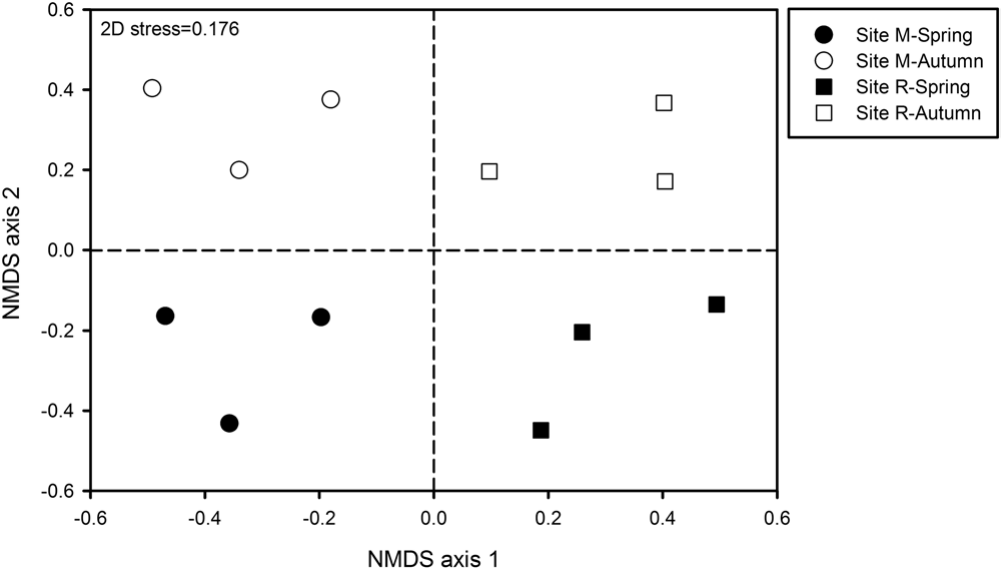
Nonmetric multidimensional scaling (NMDS) ordination of microbial functional gene community structure based on Geochip 5.0 hybridization signal intensities (using Bray-Curtis similarities) found at the deciduous forest site M (545–570 m asl) and the coniferous forest site R (1,724–1,737 m) in spring and autumn.

Richness and Shannon index of microbial functional gene community were significantly higher at coniferous forest site R respect to deciduous forest site M and significantly increased at both sites in autumn respect to spring (Table 2). According to PERMANOVA and ANOSIM analyses, functional gene structure of microbial communities significantly varied by the effect of altitude (PERMANOVA, F = 2.07, *p *= 0.0001; ANOSIM, R = 0.69, *p* = 0.0024), season (PERMANOVA, F = 1.57, *p* = 0.0019; ANOSIM, R = 0.27, *p* = 0.0393) and the interaction altitude × season (PERMANOVA, F = 1.47, *p* = 0.0035). These results were corroborated in NMDS ordination of soil samples; NMDS axis 1 separated samples of submontane site M from those of subalpine site R, while spring and autumn samples from both sites were separated by the NMDS axis 2 (Fig. 4). Altitudinal and/or seasonal differences for all the groups of functional genes, except virulence, other functions and secondary metabolism, were detected (Table S6). Among the different gene categories, genes involved in biochemical cycles of C, N, S and P as well as in organic remediation were of our particular interest. Detailed information on these gene groups is described below.

Regarding C cycling, we firstly examined the genes related to C fixation. Among the four different C fixation pathways represented in GeoChip 5.0, the Calvin cycle appeared to be the predominant one at both sites in both seasons. The genes *tktA*, *FBPase* and ribulose 1,5-bisphosphate carboxylase/oxygenase (RuBisCo) were the most abundant in this pathway over all the samples, and no significant altitudinal or seasonal shifts were detected for any of these genes (data not shown).

**Figure 5:**
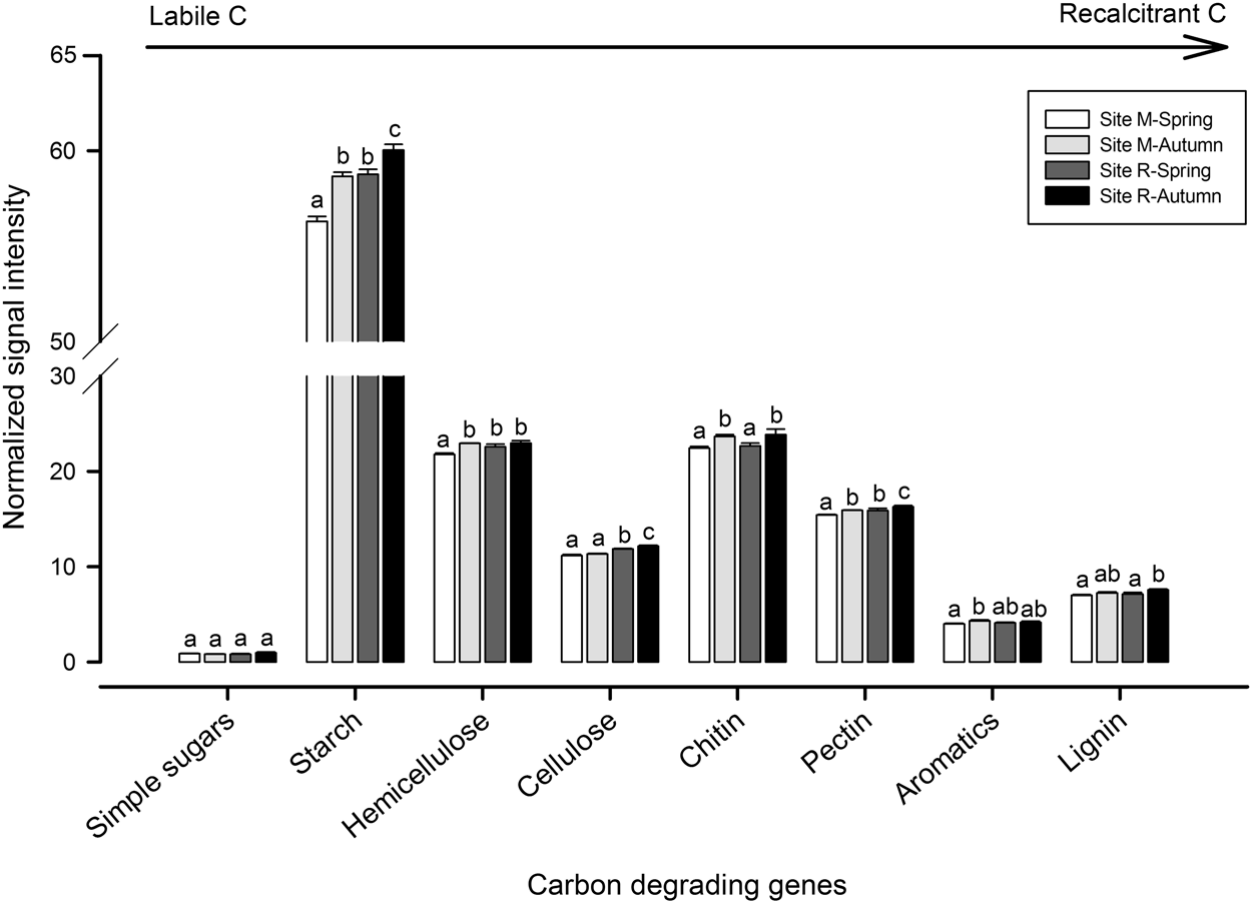
Normalized signal intensities for key C degrading genes detected using Geochip 5.0 and found at the deciduous forest site M (545–570 m asl) and the coniferous forest site R (1,724–1,737 m) in spring and autumn. The genes were grouped according to the different substrates. The targeted substrates were arranged in order from labile to recalcitrant C. For each substrate, values followed by different letters are significantly different (p ≤ 0.05) according to Tukey’s HSD test. Bars represent standard deviation.

The total abundance of genes associated with C degradation significantly increased at both sites in autumn, showing the deciduous forest site M in spring and the coniferous forest site R in autumn the significantly lowest and highest relative abundances, respectively. Approximately 40% of the total abundance of genes related to C degradation belonged to starch degradation (highlighting the abundance of the gene *amyA*, Table S7), which showed to significantly increase at both sites in autumn (Fig. 5). Significantly increases in the normalized signal intensities of the genes related to chitin (mainly chitinase genes) and pectin were observed in autumn respect to spring at both sites (Fig. 5). However, significant rises in the abundance of genes associated with hemicellulose (e.g., *ara*, xylanase, *xylA* and mannanase genes, Table S7) and aromatic compound degradation (e.g., *vanA*, *limEH*, *cdh*, *vdh*, *camDCAB* and *lmo*) in autumn were recorded only at submontane site M, while those autumn increases in genes related to cellulose (mainly genes encoding genes for cellobiase and exoglucanase enzymes, Table S7) and lignin (phenol oxidase and *mnp* genes) degradation were only recorded at subalpine site R. Except for aromatic compound - degrading genes, the abundance of genes measured at subalpine site R in autumn was always significantly higher than that of submontane site M in spring. It is worth noting that the coniferous forest site R in autumn presented significantly increased levels of genes involved in the degradation of both labile (starch) and recalcitrant (cellulose, pectin and lignin) C substrates (Fig. 5).

**Figure 6:**
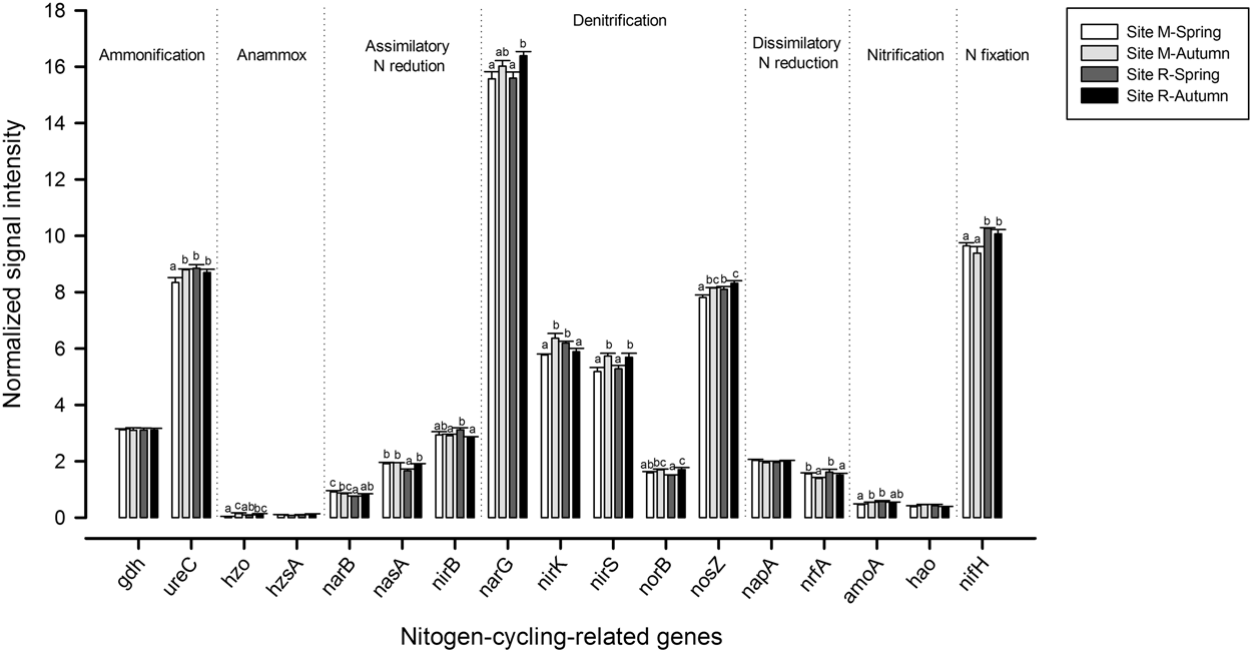
Normalized signal intensity of key genes involved in N cycling detected using Geochip 5.0 and found at the deciduous forest site M (545–570 m asl) and the coniferous forest site R (1,724–1,737 m) in spring and autumn. The genes were grouped according to their functional role in N cycling. For each gene, values followed by different letters are significantly different (p ≤ 0.05) according to Tukey’s HSD test. Statistical analyses were indicated only in genes showing significant differences. Bars represent standard deviation.

In the case of N cycling, key functional genes for ammonification, anammox, assimilatory N reduction, denitrification, dissimilatory N reduction, nitrification and N fixation were detected (Fig. 6). The gene *ureC*, involved in ammonification, showed the significantly lowest abundance at submontane site M in spring. The abundance of genes encoding proteins for denitrification process was higher in autumn at both sites except in the case of *nirK* gene. Significant differences were found for nitrification *amoA* gene at submontane site M in spring and autumn. However, no significant differences were detected for subalpine site R. The relative abundance of the gene *nifH*, related to N fixation, was significantly higher at subalpine site R and seasonal effects were not detected at any of the sites.

Regarding the S cycling, the genes related to sulfite reduction (*dsrA, dsrB* and *sir*, which catalyze the reduction of sulfite to sulfide, using iron as cofactor) were the most quantitatively abundant among all the S-cycling-related genes across all the samples, showing the coniferous forest site R in autumn the significantly highest normalized signal intensity values (Table S8). Genes encoding enzymes for sulfur oxidation showed a seasonal effect at both sites with increased levels in autumn (Table S8).

GeoChip 5.0 contained probes to target three P cycle genes (Table S8); the normalized signal intensity of *ppx* genes (encoding exopolyphosphatase enzymes for inorganic polyphosphate degradation) was higher than that of *ppk* genes (encoding polyphosphate kinase proteins for polyphosphate biosynthesis). Both groups of genes were significantly higher at subalpine site R compared to submontane site M and showed to increase in autumn at both sites (Table S8).

Among the groups of genes clustered into the organic remediation category (Table S8), the genes encoding enzymes for degradation of aromatic compounds showed to be the most abundant group. This cluster of genes, containing a high variety of probes (Table S8), showed significantly increased values in autumn at both sites, although significantly altitudinal differences were not noticed.

### **Factors driving altitudinal and seasonal changes in microbial taxonomic and functional gene communities**

According to Mantel test, altitudinal and seasonal changes in abundance, diversity and community structure of bacterial and fungal communities were mainly governed by soil pH, EC, SOM and TOC (total organic carbon) (Table S9). On the other hand, altitudinal and seasonal changes in microbial functional gene structure were significantly explained by the shifts in all the physicochemical properties determined, except NH_4_^+^-N and Mg (Table 4). TOC, SOM and soil pH were the most important variables explaining functional gene composition. The different soil temperatures tested were also significantly correlated with the changes in microbial functional gene composition as well as the abundance, the different diversity properties and the structure of bacterial community. Variations in microbial functional gene community were significantly correlated with fungal abundance and Shannon index as well as the structure of fungal community (Table 4).

**Table 4:** Mantel test results correlating microbial functional gene community structure based on Geochip 5.0 found at the deciduous forest site M (545–570 m asl) and the coniferous forest site R (1,724–1,737 m) in spring and autumn with soil physicochemical properties, soil temperature measurements and taxonomic properties of bacterial and fungal communities.

	R^2^	p-value
Soil physicochemical properties		
pH	**0.5344**	0.0017
EC^1^	**0.5048**	0.0015
SOM^2^	**0.6596**	0.0024
TOC^3^	**0.6603**	0.0023
N	**0.4514**	0.0031
NH_4_^+^-N	0.1820	0.0610
NO_3_^−^-N	**0.4617**	0.0021
C/N	**0.3154**	0.0059
P	**0.2644**	0.0155
K	**0.4881**	0.0014
Mg	0.1022	0.1016
Soil temperature		
Mean soil temperature	**0.5847**	0.0013
Maximum soil temperature	**0.5647**	0.0007
Minium soil temperature	**0.4847**	0.0010
Microbial communities		
Bacterial abundance	**0.7011**	0.0001
Bacterial richness	**0.4746**	0.0018
Bacterial Shannon index	**0.4175**	0.0048
Bacterial evenness	**0.4664**	0.0028
Bacterial community structure	**0.4968**	0.0015
Fungal abundance	**0.6932**	0.0001
Fungal richness	0.1330	0.1128
Fungal Shannon index	**0.4259**	0.0019
Fungal evenness	0.1878	0.0513
Fungal community structure	**0.4424**	0.0034

R^2^ values in bold indicate statistical significance (p ≤ 0.05).

^1^EC, electrical conductivity.

^2^SOM, soil organic matter.

^3^TOC, total organic carbon.

## Discussion

Integrative studies investigating abundance, taxonomy and functionality of soil microbial communities are important for understanding the response of microorganisms to changing environmental conditions and the ecosystem functioning^1^. In this study, we have demonstrated that soil bacterial and fungal communities from two Alpine forest sites differing in altitude and vegetation cover type, significantly differed in terms of abundance as well as taxonomic and functional gene diversity and composition (i.e., a significant altitudinal effect). However, significant seasonal changes (comparing spring and autumn) were limited to changes in functional diversity and composition (GeoChip-based) at both sites. To the best our knowledge, it is one of the first surveys where bacterial and fungal communities as well as their functional properties are assessed at the same time taking into consideration altitudinal and seasonal aspects.

According to our initial hypothesis, diversity and community structure of bacterial and fungal communities differed between the submontane forest site M and the subalpine site R, which concurs with the results of our previous work also studying these forest sites^17^. Other recent studies have also found clear soil bacterial and fungal compositional differences among different altitudes^12, 19, 32, 33^, evidencing that altitude factor is a major driver of variation in microbial composition. Since altitude is not an environmental variable itself, it has been proposed that soil microbial communities’ structure over altitudinal gradients is mainly governed by changes in vegetation cover type^13^. Plant composition influences belowground conditions by driving changes in soil environmental properties such as soil pH, litter quantity and quality, soil nutrient contents and soil moisture^34^. Conversely, the belowground community can affect the above-ground community by carrying out a wide spectrum of decomposition processes^35^. In this way, it is broadly accepted that soil physicochemical parameters served as a bridge to link the aboveground and belowground communities^26^. Therefore, we see the differences in microbial communities between both sites in connection with the different type of vegetation predominating at each forest site (the deciduous vegetation dominates at site M and coniferous one at site R) since pH as well as SOM and TOC contents showed to be the main drivers of bacterial and fungal abundance, diversity and community structure. The subalpine site R presented increased levels of C, N and other nutrients respect to the submontane site M, which can be explained by the higher recalcitrance of coniferous litter (high C/N ratio and lignin content as well as low pH^36^), producing a greater C sequestration and lower nutrient immobilization rates^17^. Furthermore, some surveys, comparing evergreen and deciduous forests, have indicated that evergreen forests accumulate more SOM in the forest floor than deciduous forests located in similar climatic zones^25^. Therefore, the increased levels of C and other nutrients detected at site R may contribute to enhance microbial growth and would explain the higher bacterial and fungal abundance detected at this forest site, as we formerly explained^17^. Likewise, the differences in SOM and other nutrient contents between both sites could explain to some extent the soil microbial composition characterizing each forest site. For example, *Gammaproteobacteria* were detected to a significantly higher extent at coniferous forest site R and the copiotrophic lifestyle of this group of bacteria has been highlighted^37^, while Actinobacteria, adapted to resource limited conditions^38^, were more abundant at deciduous forest site M. Nevertheless, it is generally recognized that the general classification of bacterial taxa as oligotrophic or copiotrophic is a clear oversimplification of large variations in ecological attributes and microbial lifestyles. In the case of fungal community, from a functional perspective, both mycorrhizal and saprotrophic fungi have shown to dominate in forest soils^28^. Both groups were indistinctly detected in the present study at both sites in spring and autumn, although they taxonomically differed between the two forest sites. Ectomycorrhizal fungi such as *Pachyphloeus* and *Amanita* predominated at deciduous forest site M while those classified as *Elaphomyces* and *Russula* dominated at coniferous forest site R. Saprotrophic fungi belonging to *Umbelopsis* genus were more abundant at submontane site M; instead, saprotrophic *Mortierella* fungi dominated at subalpine site R. In concordance, some other studies have also shown significant taxonomic differences in forest soil mycorrhizal and saprotrophic fungi in response to changes in soil depth and/or season^28^.

Network analyses are valuable tools for providing important details on community assembly rules such as cooperation, competition or habitat filtering, and to represent mathematical interactions or couplings among different microbial groups^39^. The higher average degree and shorter path distance of submontane site M network revealed that bacterial and fungal interactions were more intensive at this site, probably as a consequence of certain deterministic processes such as habitat filtering, and reflecting shared ecological tolerances. Network theory suggests that strongly connected communities are more susceptible to disturbance^40^. This assumption, along with the shorter path distance of deciduous forest site M network, indicated that microbial community at site M would respond slowly to perturbations in comparison with subalpine site R. The more susceptible community structure of submontane site M was also reflected in the lower evenness of bacterial community, which showed to positively correlate with community stability in a previous work^41^. In addition, as modularity may serve as an indicator of system resistance by compartmentalizing social-ecological systems^42^, the higher modularity of subalpine site R network indicated that the microbial system of this site would be more resistant to changes at taxonomic level. Interestingly, Ding, *et al*.^25^, also found that the soil bacterial network of a broadleaved evergreen forest presented a higher resistance to environmental changes than that of a broadleaved deciduous forest. On the other hand, we discovered that most of the OTUs identified as module hubs and connectors (generalists) by the Zi-Pi plot (Fig. S2) at both forest sites were not among the top most abundant bacterial or fungal OTUs (Tables S3 and S4), highlighting the crucial role that generalist OTUs may have as keystone species (i.e., species that play a crucial role in maintaining the structure of a community and whose impact on the community is greater than would be expected based on their relative abundance^43^). It is also worth noting that some of the generalist OTUs found in submontane site M network (OTU23B, OTU4B, OTU94B, OTU54B, OTU55B, OTU35B, OTU222F, OTU33F), were present as specialists in coniferous forest site R network, suggesting that the same OTU exhibited different roles depending on the particular environmental and soil physicochemical properties.

Contrary to our initial hypothesis, both diversity and community structure of bacterial and fungal communities did not significantly vary between spring and autumn at any of the forest sites. Our initial hypothesis was supported by one of our previous works, which showed significant spring to autumn shifts in soil PLFA-based microbial community structures of four forests over an altitudinal gradient, including the two forests studied here^30^. The differences in the significance of the factor season between both works are likely a consequence of the different methodological approaches used (PLFA analysis *vs*. Illumina amplicon sequencing) and the higher number of samples and forest sites investigated in our former study^30^. Some other surveys using amplicon sequencing for the analysis of soil microbial communities have shown a significant effect of season on diversity and composition of different forest soil bacterial^44^ and fungal^28^ communities in concomitance with significant changes in soil physicochemical properties. Therefore, in the present study, the no significant seasonal changes in the taxonomic diversity and composition of both bacterial and fungal communities is seen in connection with the lack of significant seasonal changes in most of the soil physicochemical properties tested. However, and according to Mantel test results, the trend of increased (but non-significant) SOM and nutrient contents in autumn respect to spring at both sites could explain the significant seasonal changes in the diversity and structure of microbial functional gene community observed at both sites. These findings would be supporting the idea that soil microbial communities of these sites are able to significantly respond in functional terms to subtle shifts in environmental conditions. However, more drastic differences in environmental conditions (as those observed between different altitudes) are needed to detect significant differences in microbial communities in abundance and taxonomic terms. In this regard, Burke, *et al*.^45^ suggested that microbial responses to environmental shifts may be better predicted by functional genes rather than taxonomy.

The relationships between taxonomic and functional diversity in soils are largely unknown^46^. Several recent surveys are focused on finding these relationships at different ecosystem scales by combining bacterial and fungal ribosomal gene sequencing with whole genome shotgun sequencing or GeoChip analyses^22^. For example, Fierer, *et al*.^47^ showed a strong positive correlation between taxonomic and functional gene diversity in the tallgrass prairie soils of the midwestern United States, suggesting a low degree of functional redundancy. Likewise, Zhang, *et al*.^48^, comparing the soil microbial communities of a natural mature forest with those of a natural secondary forest, found a strong positive correlation between the soil bacterial community structure and the GeoChip-based functional community composition. However, there are other studies showing no significant correlations between microbial taxonomic and functional composition^25, 39^, which is explained as a consequence of the functional redundancy of soil microbial communities^49^. The contradictory results may be caused by differences in terms of techniques, scale or ecosystem investigated^22^. In our study, although Mantel test results showed that microbial functional gene composition significantly correlated with taxonomic diversity and structure of bacterial and fungal communities, the no concomitant seasonal changes in microbial taxonomy and function may be related to the aforementioned functional redundancy of soil microbial communities. In fact, when Mantel tests are repeated using only the data for each site in spring and autumn, significant correlations are not found. Functional redundancy of species is recognized as a common feature in soils^49^, which is based on findings revealing that some species carry out similar roles in communities and/or ecosystems^46^. However, functional redundancy is difficult to establish because it requires detailed information about the microbial populations that perform a specific process. Furthermore, microorganisms that are functionally redundant under one set of conditions may not be under other different conditions. In general, our knowledge about the distribution of functional traits across microbial taxa is still scarce^46^. Therefore, more studies using multiple comparable techniques are needed in the future to test taxonomic and functional relationships across different ecosystems.

The quantity and quality of available C and N in soil are important regulating soil microbial-mediated processes and functions^50^. For example, the degradation of highly humified SOM has shown to be energy limited, determining very slow SOM decomposition rates in soils. However, the input of labile C may provide fresh energy and enable microorganisms to degrade SOM more efficiently^51^. Complementarily, former studies have also suggested that changes in soil N content and availability determine alterations in rates of microbial-mediated SOM and litter decomposition. Altogether, these findings evidence that the availability of labile C and N controls the decomposition of complex substrates and thus the availability of nutrients for plants^50^. In this context, in our study, the lack of altitudinal or seasonal shifts in the genes related to the major C fixation pathway (Calvin cycle), on one hand, and the predominance of genes associated with C degradation among those related to C-cycling, on the other hand, could be evidencing a dependency of the soil microorganisms from these two forest ecosystems to external C inputs (e.g., rhizodeposits and exudates, dead roots or litter). We see this finding in line with the significantly increased signal intensities of C degrading genes and the trend of higher soil C contents detected in autumn respect to spring at both sites. Autumn is characterized by the leaching of nutrients and compounds from the upper soil horizons after litterfall and/or the deposition of easily decomposable compounds from roots in soil after the end of the vegetation period, as it has been discussed in our former article^30^. It is worth noting that coniferous forest site R in autumn presented significantly increased levels of genes encoding enzymes for the degradation of both labile and recalcitrant C sources, which is seen in connection with the results of the network analysis showing a more resistant microbial community to environmental changes at this site and with the significantly higher potential β-glucosidase, xylosidase and cellobiohydrolase activities detected at this site respect to site M in one of our previous works investigating these forest sites^30^.

Regarding the Geochip-based analysis of N-cycling genes, it is interesting to note that we did not find a significant correlation between the total abundances of nitrification genes (*amoA* and *hao*) and NH_4_^+^-N (R = 0.152, *p* = 0.152), nor between the abundance of denitrification genes (*nirS* and *nirK*) and NO_3_^−^-N (R = 0.311, *p* = 0.124), suggesting a decoupling between the gene signals detected and the real activity of microorganisms. In this context, it is important to bear in mind that GeoChip microarray data reflect potential functional capacity in the environment^52^.

In conclusion, we here demonstrated that altitude, comparing two Alpine forest sites located at two different altitudes and characterized by different vegetation cover types (deciduous forest *vs*. coniferous forest), soil physicochemical properties and soil temperatures, had a strong significant effect on abundance, taxonomic diversity and structure, and network structure of soil bacterial and fungal communities, as well as on soil microbial functional gene diversity and composition. However, season (comparing spring and autumn) only exerted a significant effect on functional gene diversity and composition. Altitudinal and seasonal changes in abundance, diversity and community structure of bacterial and fungal communities as well as in microbial functional gene composition were mainly explained by variations in soil pH, SOM and nutrient contents as well as in soil temperatures. Therefore, the detection of only significant seasonal changes in microbial functional diversity and community structure is likely a consequence of the non-significant increased SOM and nutrient contents detected in autumn respect to spring at both forest sites and the presumable high ability of soil microbial communities to respond in functional terms to discreet environmental changes. In the future, further studies, considering forests in a wider range of altitudes and the four seasons of the year, are needed to deepen our understanding regarding the effect of the changing environmental conditions related to shifts in altitude and season on soil microbial communities.

## Methods

### Study sites

The two investigated sampling sites M (Montiggl) and R (Ritten) are two long-term monitoring sites, which were installed in the Italian Alps in 1992 within the framework of ICP IM (international cooperative programme on integrated monitoring of air pollution effects on ecosystems)^53^. These sites represent two widely distributed and forestally significant forest types in South Tyrol (Italy). A detailed description of each site has previously been reported^29, 30, 54^. Briefly, the submontane site M (N 46° 25′ 36.8″, E 11° 17′ 48.6″) is located 8 km south of Bozen/Bolzano on the small peak Kleiner Priol at an altitude of 545-570 m asl. The pedogenic substratum consists of rhyolite (quartz-porphyry) and the soil was classified as dystric cambisol (FAO). The site consists of mixed deciduous forest, dominated by *Quercus pubescens*, *Q. robur*, *Fraxinus ornus, Pinus sylvestris* and *Ostrya carpinifolia*. The climate is mild continental with submediterranean influences, with a mean annual air temperature of 11.0 °C and an annual precipitation of 900 mm.

The subalpine site R (N 46° 35′ 16.2″, E 11° 26′ 4.9″) is located 7 km north of Bozen/Bolzano below the Rittner Horn at an altitude of 1,724-1,737 m asl. The pedogenic substratum consists of rhyolite (quartz-porphyry) and the soil was classified as haplic podzol (FAO). The site consists of coniferous forest close to the timber line, dominated by *Picea abies*, *P. cembra*, *Larix decidua* and *Vaccinium myrtillus*. The climate is subalpine-continental with a mean annual air temperature of 4.0 °C and an annual precipitation of 1000 mm.

### Soil sampling

Six sampling spots, distributed uniformly over each site (100 × 100 m) and considered as independent replicates, were chosen to cover within-site variability. Soil samples were collected from each of these sampling spots from the A_h_ horizon (top 10 cm); the number of sampled cores (2–5) depended on the thickness of the sampled A_h_ horizon at each site^30^. The distance between sampling spots in each sampling area was site-dependent^17^. To determine the effect of season at each site and to take into account the different vegetation periods at the investigated sites, soil samples were collected both in late spring (site M: 15 April 2015; site R: 18 May 2015) and autumn (site M: 30 October 2015; site R: 5 October 2015). The 24 soil samples (6 samples × 2 sites × 2 seasons) were transported immediately after sampling in cooled boxes to the laboratory, sieved (2 mm mesh) and stored at 4 °C for physicochemical characterization or at −80 °C prior to DNA extraction.

### Soil physicochemical characterization and soil temperature

Each soil sample was characterized regarding soil pH, electrical conductivity (EC), SOM, total organic carbon (TOC), total nitrogen (N), C/N (TOC/N) ratio as well as plant available P, K and Mg, as formerly described^30^. Inorganic N forms, NH_4_^+^-N and NO_3_^−^-N, were measured in 1 M KCl soil extracts, as reported by Rhine, *et al*.^55^ and Cataldo, *et al*.^56^, respectively.

Soil temperature was measured in triplicate at each site using DS1921G Thermochron iButton dataloggers buried at a depth of ca. 5 cm in the A_h_ horizon (DS1921G-F5eve#, Maxim Semiconductor Inc.).

### DNA extraction

Total DNA from each soil sample (24) was extracted in triplicate from 250 mg of soil fresh mass using Power Soil™ DNA Isolation Kit (MO BIO Laboratories Inc., Solana Beach, USA) following the manufacturer’s instructions. Triplicate DNA extracts from each soil sample were then pooled, and DNAs were purified using DNA Clean & Concentrator Kit (Zymo Research, Irvine, USA) according to manufacturer’s instructions. The quality of DNAs was spectrophotometrically checked by NanoDrop (Thermo Fisher Scientific Inc., Bremen, Germany) based on the absorbance ratios A_260_/A_280_ and A_260_/A_230_. Subsequently, the DNAs were quantified using QuantiFluor™ dsDNA System (Promega, Madison, USA) and DNA concentration for each sample was standardized to 20 ng μL^−1^.

### Quantitative PCR analyses

The quantification of bacterial 16S rRNA and fungal 18S rRNA genes was conducted by qPCR from each DNA sample (24) with the pair of primers Eub338 /Eub518^57^ for bacteria and FR1/FF390^58^ for fungi using SYBR^®^ Green as detection system (Bio-Rad, Hercules, USA) under the PCR mixtures and thermal conditions previously described^17^. Bacterial and fungal standard curves were generated using a recombinant plasmid containing one copy of 16S rRNA and 18S rRNA genes, respectively, as described by Siles & Margesin^17^. The curves were drawn by plotting the Ct values as a log function of the copy number of 10-fold serial dilutions of the plasmid DNA. The relationship between Ct and the target-gene copy number, on the one hand, and the copy numbers of the real-time standard, on the other, were calculated as previously described^59^. The copy numbers of bacterial 16S rRNA and fungal 18S rRNA genes were expressed as copy number per gram dry mass soil.

### 16S rRNA gene fragment and Internal Transcribed Spacer (ITS) 1 sequencing

Prokaryotic communities were characterized amplifying the V4-V5 hypervariable region of 16S rRNA gene using the primers new515F (5′-GTGYCAGCMGCCGCGGTAA-3′) and 909 R (5′-CCCCGYCAATTCMTTTRAGT-3′)^60, 61^. From each site and season, six DNA samples were amplified in triplicate using HotStarTaq Plus Master Mix Kit (Qiagen, Valencia, USA) containing barcoded forward primers, under the next thermal conditions: initial denaturation at 94 °C for 3 minutes, followed by 28 cycles of denaturation at 94 °C for 30 s, primer annealing at 53 °C for 40 s and extension at 72 °C for 60 s as well as a final elongation step at 72 °C for 5 minutes. The success of the amplification was checked in a 2% agarose gel and reactions of the same sample were merged. Subsequently, PCR products from all the samples were purified using Agencourt AMPure XP magnetic beads kit (Beckman Coulter, Inc., Pasadena, USA), quantified with the QuantiFluor™ dsDNA System and pooled in equal proportions. The pooled product was then used to prepare Illumina DNA library following TruSeq DNA library preparation protocol. Paired-end sequencing (2 × 300) was performed on the Illumina MiSeq sequencing platform (Illumina, San Diego, USA) at MR DNA (www.mrdnalab.com, Shallowater, TX, USA).

Fungal communities were analysed by amplification of ITS1 region using the ITS1F (5′-CTTGGTCATTTAGAGGAAGTAA-3′) and ITS2 (5′-GCTGCGTTCTTCATCGATGC-3′) primers^62, 63^, with the next cycling conditions: 94 °C for 3 minutes, followed by 28 cycles of 94 °C for 30 s, 57 °C for 40 s and 72 °C for 60 s, and a final extension of 72 °C for 5 minutes. The processing of amplicons for paired-end sequencing (2 × 300) on Illumina MiSeq sequencing platform was as described for prokaryotic community.

The raw bacterial and fungal sequences associated with this study were deposited in the GenBank SRA database under BioProject accession number PRJNA378480.

### Bioinformatic and diversity analyses

First, raw MiSeq paired-end reads from prokaryotic 16 rRNA and fungal ITS amplicons were separately assembled and reoriented using MR DNA pipeline. Subsequently, sequences were demultiplexed and formatted for processing using a Phython script^17^. Next, sequences from each library were processed using USEARCH pipeline and UPARSE-OTU algorithm^64^. Briefly, reads were separately quality-filtered allowing a maximum e-value of 1 for both libraries, trimmed (to 350-bp (base pair) and 240-bp for prokaryotic and fungal libraries, respectively), dereplicated and sorted by abundance (removing singleton sequences) prior OTU determination at 97% sequence identity. Then, chimeric representative sequences from the OTUs were detected and removed using UCHIME^65^. Finally, original high-quality sequences were mapped to OTUs at the 97% identity threshold obtaining one OTU table for prokaryotic community and another one for fungal community. The taxonomic affiliation of each OTU was obtained using RDP (ribosomal database project) taxonomic classifier against 16S rRNA training set 14 for prokaryotic sequences and UNITE Fungal ITS train set 07-04-2014 for fungal sequences with a 50% confidence threshold for both cases. Next, OTUs classified as Archaea were removed from prokaryotic OTU table. All the remaining prokaryotic OTUs and all the OTUs from fungal dataset could be classified as Bacteria and Fungi, respectively, and they were thus retained. These two OTU tables were used for downstream analyses.

For diversity characterization of microbial communities and statistical analyses, the number of sequences per sample was normalized to 25,000 sequences for bacteria and 40,000 sequences for fungi. The bacterial and fungal communities were characterized in terms of diversity by calculating richness (number of OTUs), Shannon index, Smith-Wilson evenness and the richness estimator indices ACE (abundance-based coverage estimation) and Chao 1 using Mothur v.1.37.0^66^.

### GeoChip analysis

The small version of GeoChip 5.0 (containing near of 60,000 gene probes) was used for functional characterization of soil microbial communities at both sites in spring and autumn. Three DNA samples per site and season were analyzed. To do this, the original six DNAs extracts from each site and season were merged two by two to give three pooled pairs of composite samples. GeoChip analyses were done as previously described^67^. Briefly, DNA from each sample (~1 µg) was firstly labeled with the fluorescent nucleic acid dye Cy3 using a random priming method. The labeled DNA was subsequently purified and dried before hybridization on Agilent platform-based GeoChip 5.0 arrays at 67 °C for 24 h. GeoChip microarrays were then scanned with a SureScan Microarray Scanner (Agilent Technologies, Santa Clara, CA). Raw data were quantified and normalized as previously described^67^. Spots with a signal-to-noise ratio <2.0, signals <1.3 times the background or <1000 were discarded. Additionally, spots that were detected in less than two samples (either within a replicate group or across all samples) were also removed. Before statistical analyses, data were: (i) normalized by dividing the signal of each spot by the total intensity of the microarray; (ii) multiplied by a constant; and (iii) transformed by the natural logarithm. Microbial functional gene diversity was characterized by calculating richness (number of detected genes), Shannon index and Smith-Wilson evenness using Mothur v.1.37.0.

### Network analysis

A network analysis was carried out to compare the soil microbial network structures of two altitude-contrasting forest sites and identify key bacterial and fungal OTUs at both sites. One network analysis per site was carried out. To do this, the bacterial and fungal OTU tables from each forest site in both seasons were firstly merged. Bacterial or fungal OTUs detected in less than 10 of 12 replicates at each forest site were removed to ensure reliable correlation networks. Datasets were then uploaded to the molecular ecological network analysis pipeline (MENAP, http://ieg2.ou.edu/MENA) and the networks were constructed using random matrix theory-based methods and default parameters. More information on theories, algorithms, pipeline structure and procedures was described by Zhou, *et al*.^68, 69^ and Deng, *et al*.^70^. An identical similarity threshold (0.900) for both sites was chosen for network constructions to allow direct comparisons. To characterize the modularity property, each network was separated into modules by the fast greedy modularity optimization. Various indices were calculated and used to descript and compare topological properties of networks at each forest site (see Table 3 for more details regarding the selected indices). Topological roles of individual nodes (OTUs) in the networks were evaluated by the threshold values of Zi (within-module connectivity) and Pi (among-module connectivity). The threshold values of Zi and Pi for categorizing OTUs were 2.5 and 0.62, respectively^71^. Networks were graphed using Cytoscape v. 3.4.0. Small modules with less than 10 nodes were ignored for network graphic representation.

### Statistical analyses

One-way analysis of variance (ANOVA) was applied to determine whether there were significant differences (p ≤ 0.05) between the sites M and R in spring and autumn regarding (i) soil physicochemical properties; (ii) soil temperature; (iii) bacterial and fungal abundance; (iv) bacterial, fungal and microbial functional diversity measurements; (v) relative abundance of the different taxonomic bacterial and fungal groups; (vi) relative abundance of the top most abundant bacterial and fungal OTUs; and vii) normalized signal intensities of the different functional groups detected using GeoChip 5.0. Tukey’s honest significance difference (HSD) post-hoc test was used for multiple comparisons of means at a 95% confidence interval. Normality and heteroscedasticity of data were tested by the Shapiro-Wilk and Levene tests, respectively. In case that one of those conditions was not met, the values were log transformed.

The significance of the factors altitude and season, on the structure of bacterial, fungal and microbial functional gene communities, was tested using permutational analysis of variance (PERMANOVA) and analysis of similarity (ANOSIM) tests with Bray-Curtis similarities and 9999 permutations. Non-metric multidimensional scaling (NMDS) analysis based on Bray-Curtis similarities at OTU level was used to visualize patterns in bacterial and fungal community structures. NMDS analysis was also applied for GeoChip 5.0 dataset. All the analyses were done using “vegan” package in R^72^.

Mantel test, comparing Bray-Curtis similarities matrices with 9,999 permutations, was used to detect significant correlations (p ≤ 0.05) between microbial functional gene community composition and the different physicochemical properties tested, soil temperature measurements as well as bacterial and fungal abundances. Mantel test was also used to study the relationships between abundance, diversity (Shannon index) and community structure of bacterial and fungal communities and the different soil physiochemical properties and soil temperatures. “Vegan” package in R was used for Mantel tests.

## Electronic supplementary material

Supplementary Information
