# Supplementary material for: Seasonal soil microbial responses are limited to changes in functionality at two Alpine forest sites differing in altitude and vegetation
Source: Sci Rep. 2017 May 19;7:2204. doi: 10.1038/s41598-017-02363-2 (PMC5438347; doi:10.1038/s41598-017-02363-2)

SUPPLEMENTARY INFORMATION

Seasonal soil microbial responses are limited to changes in functionality at two Alpine forest sites differing in altitude and vegetation

José A. Siles*, **Rosa Margesin**

*corresponding author: José A. Siles; Email: jose.siles-martos@uibk.ac.at

Scientific Reports

The supplementary information includes Tables S1-S9 and Figures S1-S2

Table S1. Relative abundance (%) of the most abundant bacterial phyla and classes found at the deciduous forest site M (545-570 m asl) and the coniferous forest site R (1,724-1,737 m) in spring and autumn. For each taxonomic group, values followed by different letters are significantly different (p≤0.05) according to Tukey's HSD test.

	Site M		Site R	
	Spring	Autumn	Spring	Autumn
**Bacterial phyla**				
Proteobacteria	31.67 a	32.89 a	31.99 a	31.54 a
Acidobacteria	22.38 a	23.28 ab	36.08 bc	39.08 c
Bacteroidetes	16.52 b	12.56 ab	8.97 a	8.40 a
Actinobacteria	10.04 b	9.62 b	4.27 a	3.66 a
Verrucomicrobia	7.83 ab	8.22 b	5.20 ab	5.14 a
Planctomycetes	3.62 a	3.49 a	3.32 a	2.72 a
WPS-2	1.22 a	1.46 a	2.58 a	2.11 a
Unclassified	2.61 a	3.70 a	3.87 a	3.38 a
Others (18)	4.12 a	4.77 a	3.72 a	3.96 a
**Bacterial classes**				
Alphaproteobacteria (*Proteobacteria*)	19.20 b	21.02 b	15.33 ab	14.04 a
Gp 1 (*Acidobacteria*)	7.52 a	7.27 a	17.33 b	16.68 b
Sphingobacteriia (*Bacteroidetes*)	11.56 a	9.57 a	7.64 a	7.87 a
Gp 2 (*Acidobacteria*)	4.63 a	3.88 a	13.44 ab	13.22 b
Gammaproteobacteria (*Proteobacteria*)	4.52 a	4.03 a	9.53 b	10.71 b
Actinobacteria (Actinobacteria)	10.52 b	9.88 b	3.96 a	3.62 a
Gp 3 (*Acidobacteria*)	4.76 a	5.19 ab	7.23 ab	7.58 b
Betaproteobacteria (*Proteobacteria*)	4.47 b	5.22 b	1.74 a	2.11 ab
Planctomycetia (*Planctomycetes*)	3.76 a	3.75 a	3.43 a	2.73 a
Spartobacteria (*Verrucomicrobia*)	5.49 b	6.35 b	1.01 a	0.91 a
Deltaproteobacteria (*Proteobacteria*)	2.53 a	3.48 a	3.05 a	3.10 a
Subdivision 3 (*Verrucomicrobia*)	1.30 a	1.36 a	2.67 b	2.91 b
Incertae sedis (WPS2)	1.32 a	1.50 ab	2.06 b	1.96 ab
Gp 6 (*Acidobacteria*)	1.25 bc	1.84 c	0.31 a	0.28 ab
Opitutae (*Verrucomicrobia*)	0.55 a	0.72 a	1.10 a	0.90 a

Table S2. Identification and relative abundances of bacterial OTUs with abundances ≥0.25% considering the total number of sequences from the 24 libraries found at the deciduous forest site M (545-570 m asl) and the coniferous forest site R (1,724-1,737 m) in spring and autumn. For each bacterial OTU, values followed by different letters are significantly different (p≤0.05) according to Tukey's HSD test.

		Site M		Site R	
OTU no.	OTU identification, (Class/Subgroup), % confidence threshold	Spring	Autumn	Spring	Autumn
OTU3	*Povalibacter* (*Gammaproteobacteria*), 97	4.42 a	3.13 a	10.63 b	11.33 b
OTU1	*Bradyrhizobium* (*Alphaproteobacteria*), 100	9.98 b	10.59 b	3.33 a	2.87 a
OTU4	Gp 1 (*Acidobacteria*), 98	3.30 a	3.00 a	6.42 ab	8.17 b
OTU8	*Ferruginibacter* (*Sphingobacteriia*), 66	3.66 a	2.68 a	5.51 a	4.62 a
OTU2	Gp 2 (*Acidobacteria*), 100	0.34 a	0.24 a	6.22 b	5.47 b
OTU29	*Rhodoplanes* (*Alphaproteobacteria*), 51	1.03 a	1.02 a	4.07 b	3.23 b
OTU9	*Terriglobus* (*Acidobacteria*), 65	2.13 a	1.72 a	2.77 a	2.46 a
OTU12	*Mycobacterium* (*Actinobacteria*), 100	3.86 b	4.15 b	0.76 a	0.71 a
OTU18	Spartobacteria_genera_incertae_sedis (*Spartobacteria*), 100	4.28 b	5.16 b	0.10 a	0.06 a
OTU5	Gp 2 (*Acidobacteria*), 100	0.58 a	0.52 a	2.53 b	2.85 b
OTU10	Gp 2 (*Acidobacteria*), 100	0.27 a	0.17 a	1.82 a	3.73 b
OTU37	Spartobacteria_genera_incertae_sedis (Spartobacteria), 100	3.51 b	4.59 b	0.09 a	0.05 a
OTU6	*Aggregicoccus* (*Deltaproteobacteria*), 16	0.48 a	0.52 a	2.95 a	2.01 a
OTU7	*Roseiarcus* (*Alphaproteobacteria*), 99	1.16 a	1.17 a	2.09 b	2.07 b
OTU15	Gp 1 (*Acidobacteria*), 99	0.87 a	0.96 a	2.21 a	1.70 a
OTU48	*Rhizomicrobium* (*Alphaproteobacteria*), 59	2.28 a	2.86 a	0.99 a	0.91 a
OTU76	Gp 2 (*Acidobacteria*), 100	0.80 ab	0.51 a	1.72 ab	2.44 b
OTU26	Candidatus Solibacter (*Acidobacteria*), 99	1.29 a	1.39 a	1.35 a	1.68 a
OTU23	Gp 3 (*Acidobacteria*), 98	0.83 a	0.87 a	1.73 b	1.70 b
OTU31	*Telmatobacter* (*Acidobacteria*), 81	0.53 a	0.45 a	1.84 b	2.10 b
OTU11	*Flavitalea* (*Sphingobacteriia*), 43	2.62 a	2.12 a	0.56 a	0.80 a
OTU94	Gp 2 (*Acidobacteria*), 100	2.23 b	2.55 b	0.41 a	0.52 a
OTU133	Gp 6 (*Acidobacteria*), 100	1.78 ab	3.26 b	0.51 a	0.63 a
OTU53	*Chitinophaga* (*Sphingobacteriia*), 33	1.10 a	1.79 a	1.59 a	0.91 a
OTU30	*Burkholderia* (*Betaproteobacteria*), 99	0.87 a	0.84 a	1.17 a	1.88 a
OTU22	*Acidipila* (Gp 1, *Acidobacteria*), 98	0.72 a	0.50 a	1.78 b	1.56 b
OTU56	*Rhodoplanes* (*Alphaproteobacteria*), 30	1.90 b	2.49 b	0.61 a	0.51 a
OTU17	*Mucilaginibacter* (*Sphingobacteriia*), 100	2.53 a	3.30 a	0.28 a	0.23 a
OTU28	Candidatus *Solibacter* (Gp 3, *Acidobacteria*), 92	0.49 a	0.60 a	1.55 b	1.63 b
OTU24	*Actinoallomurus* (*Actinobacteria*), 84	1.28 a	1.57 a	0.87 a	0.99 a
OTU32	*Mucilaginibacter* (*Sphingobacteriia*), 100	1.16 a	0.62 a	1.35 a	1.25 a
OTU14	Gp 3 (*Acidobacteria*), 100	0.68 a	0.64 a	1.32 b	1.32 b
OTU16	Gp 1 (*Acidobacteria*), 100	0.58 a	0.69 a	0.93 a	1.68 a
OTU280	*Rhodoplanes* (*Alphaproteobacteria*), 69	0.80 a	0.97 a	1.25 a	1.09 a
OTU144	Genera_incertae_sedis (Subdivision 3, Verrucomicrobia), 100	0.23 a	0.16 a	1.68 b	1.43 b
OTU1346	Gp 2 (*Acidobacteria*), 100	1.51 b	2.02 b	0.37 a	0.23 a
OTU21	Gp 1 (*Acidobacteria*), 100	0.20 a	0.21 a	1.70 b	1.23 b
OTU41	*Steroidobacter* (*Gammaproteobacteria*), 40	0.77 a	1.02 a	0.77 a	1.22 b
OTU40	*Mucilaginibacter* (*Sphingobacteriia*), 70	2.47 a	1.14 a	0.43 a	0.36 a
OTU43	Gp 3 (*Acidobacteria*), 52	1.32 b	1.58 b	0.51 a	0.58 a
OTU121	Gp 2 (*Acidobacteria*), 100	1.59 b	1.24 ab	0.46 a	0.53 a
OTU27	*Permianibacter* (*Gammaproteobacteria*), 16	0.31 a	0.19 a	1.18 b	1.37 b
OTU77	*Rhizomicrobium* (*Alphaproteobacteria*), 61	1.03 ab	1.38 b	0.68 ab	0.58 a
OTU46	Gp 1 (*Acidobacteria*), 86	0.44 a	0.54 a	1.06 b	1.03 b
OTU13	Gp 1 (*Acidobacteria*), 100	0.37 ab	0.26 a	0.85 ab	1.13 b
OTU117	*Nitrosococcus* (*Gammaproteobacteria*), 28	0.62 a	0.52 a	0.76 a	0.97 a
OTU45	*Mucilaginibacter* (*Sphingobacteriia*), 99	2.12 a	1.01 a	0.21 a	0.19 a
OTU85	Gp 1 (*Acidobacteria*), 100	1.15 b	1.44 b	0.29 a	0.25 a
OTU3807	Gp 2 (*Acidobacteria*), 100	0.40 ab	0.21 a	0.86 ab	1.00 b
OTU4335	Gp 3 (*Acidobacteria*), 100	0.99 a	1.14 a	0.33 a	0.42 a
OTU50	*Acidisoma* (*Alphaproteobacteria*), 36	1.06 a	0.64 a	0.64 a	0.56 a
OTU3213	*Mucilaginibacter* (*Sphingobacteriia*), 97	1.90 a	0.73 a	0.37 a	0.19 a
OTU97	*Actinoallomurus* (*Actinobacteria*), 80	0.55 a	0.68 ab	0.84 b	0.68 ab
OTU20	Gp 3 (*Acidobacteria*), 82	0.93 b	1.09 b	0.44 a	0.46 a
OTU57	*Acidisoma* (*Alphaproteobacteria*), 75	0.89 a	0.72 a	0.61 a	0.54 a
OTU55	WPS-2 (WPS-2), 100	0.11 a	0.06 a	1.21 b	0.98 b
OTU138	*Limisphaera* (Subdivision 3), 41	0.18 a	0.10 a	1.12 b	1.02 b
OTU63	*Thermomonospora* (*Actinobacteria*), 13	0.53 a	0.27 a	1.16 a	0.56 a
OTU51	Subroup 1 (*Acidobacteria*), 100	1.30 b	1.59 b	0.02 a	0.02 a
OTU65	*Chitinophagaceae* (*Sphingobacteriia*), 100	1.45 a	0.69 a	0.45 a	0.26 a
OTU25	*Asanoa* (*Actinobacteria*), 48	1.67 b	1.64 b	0.02 a	0.01 a
OTU216	*Telmatobacter* (Gp 1, *Acidobacteria*), 98	0.60 a	0.43 a	0.71 a	0.60 a

OTU39	*Variibacter* (*Alphaproteobacteria*), 38	0.97 b	1.32 b	0.24 a	0.22 a
OTU131	*Labilithrix* (*Deltaproteobacteria*), 69	0.17 a	0.23 a	0.88 b	0.83 b
OTU177	Gp 1 (*Acidobacteria*), 100	0.19 a	0.30 a	0.85 b	0.78 b
OTU79	*Flavobacterium* (*Flavobacteriia*), 100	1.76 a	1.01 a	0.23 a	0.03 a
OTU124	Genera_incertae_sedis (Subdivision 3, *Verrucomicrobia*), 100	0.42 ab	0.29 a	0.65 bc	0.83 c
OTU35	Gp 2 (*Acidobacteria*), 100	0.69 a	0.51 a	0.59 a	0.47 a
OTU42	*Nitrospirillum* (*Alphaproteobacteria*), 33	0.89 b	1.21 b	0.20 a	0.21 a
OTU3862	Gp 1 (*Acidobacteria*), 99	0.08 a	0.05 a	0.96 b	0.85 b
OTU113	*Conexibacter* (*Actinobacteria*), 87	0.61 a	0.62 a	0.54 a	0.42 a
OTU294	*Xanthobacter* (*Alphaproteobacteria*), 27	0.47 a	0.54 a	0.60 a	0.50 a
OTU66	Genera_incertae_sedis (*Spartobacteria*), 100	0.75 b	0.86 b	0.26 a	0.31 a
OTU71	*Opitutus* (*Opitutae*), 100	0.44 a	0.56 a	0.55 a	0.53 a
OTU156	*Tepidimonas* (*Betaproteobacteria*), 51	0.89 b	1.60 b	0.05 a	0.02 a
OTU62	Gp 1(*Acidobacteria*), 100	0.66 a	0.60 a	0.33 a	0.41 a

Table S3. Relative abundance (%) of the fungal phyla and the most abundant fungal orders found at the deciduous forest site M (545-570 m asl) and the coniferous forest site R (1,724-1,737 m) in spring and autumn. For each taxonomic group, values followed by different letters are significantly different (p≤0.05) according to Tukey's HSD test.

	Site M		Site R	
	Spring	Autumn	Spring	Autumn
**Fungal phyla**				
Basidiomycota	57.14 a	52.67 a	52.81 a	43.32 a
Ascomycota	29.48 a	28.67 a	41.59 a	46.27 a
Zygomycota	3.91 a	3.54 a	2.31 a	2.25 a
Others	0.34 a	0.34 a	0.16 a	0.11 a
Unclassified	9.12 a	14.77 a	3.14 a	8.04 a
**Fungal orders**				
Eurotiales (*Ascomycota*)	15.97 ab	3.73 a	29.03 b	36.16 b
Agaricales (*Basidiomycota*)	28.79 b	18.55 ab	14.33 a	21.39 ab
Russulales (*Basidiomycota*)	10.92 a	10.90 a	16.22 a	12.87 a
Atheliales (*Basidiomycota*)	3.06 a	1.66 a	13.59 a	4.52 a
Thelephorales (*Basidiomycota*)	5.35 bc	8.87 c	1.77 ab	0.71 a
Cantharellales (*Basidiomycota*)	0.60 a	4.64 a	4.57 a	2.08 a
Helotiales (*Ascomycota*)	2.54 a	5.02 a	3.59 a	1.56 a
Pezizales (*Ascomycota*)	4.55 b	7.47 b	0.17 a	0.19 a
Mortierellales (*Zygomycota*)	1.54 a	1.47 a	2.19 a	2.16 a
Chaetothyriales (*Ascomycota*)	1.84 ab	4.96 b	1.45 ab	0.45 a
Unidentified *Ascomycota* (*Ascomycota*)	0.84 a	2.06 a	1.18 a	1.34 a
Boletales (*Basidiomycota*)	1.58 a	3.05 a	0.53 a	0.53 a
Mucorales (*Zygomycota*)	2.28 b	1.98 b	0.11 a	0.09 a
Unidentified Eurotiomycetes (*Ascomycota*)	1.10 a	1.81 a	0.04 a	0.04 a
Hysteriales (*Ascomycota*)	0.98 b	1.15 b	0.24 a	0.35 a
Incertae_sedis_8 (*Ascomycota*)	0.13 a	0.11 ab	0.43 ab	1.16 b
Geminibasidiales (*Basidiomycota*)	1.41 b	1.75 b	0.12 a	0.11 a
Unidentified Pezizomycetes (*Ascomycota*)	0.03 a	0.02 a	0.68 a	0.55 a
Unidentified Agaricomycetes (*Basidiomycota*)	1.15 a	0.78 a	0.09 a	0.06 a
Sebacinales (*Basidiomycota*)	0.91 ab	0.39 ab	0.19 ab	0.07 a

Table S4. Identification and relative abundances of fungal OTUs with abundances ≥0.25% considering the total number of sequences from the 24 libraries found at the deciduous forest site M (545-570 m asl) and the coniferous forest site R (1,724-1,737 m) in spring and autumn. For each OTU, values followed by different letters are significantly different (p≤0.05) according to Tukey's HSD test.

		Site M		Site R	
OTU no.	OTU identification (Class, Domain), % confidence threshold	Spring	Autumn	Spring	Autumn
OTU1	*Elaphomyces* (*Eurotiales*, *Ascomycota*), 100	0.47 a	0.40 a	17.40 b	28.98 b
OTU2	*Elaphomyces* (*Eurotiales*, *Ascomycota*), 100	5.78 a	2.41 a	13.32 a	3.17 a
OTU4	*Russula* (*Russulales*, *Basidiomycota*), 100	0.34 a	0.36 a	14.18 b	6.20 b
OTU3	*Cortinarius* (*Agaricales*, *Basidiomycota*), 100	0.15 a	0.16 a	2.11 a	11.65 a
OTU6	*Amanita* (*Agaricales*, *Basidiomycota*), 100	9.47 b	11.54 b	0.06 a	0.07 a
OTU7	*Piloderma* (*Atheliales*, *Basidiomycota*), 100	0.06 a	0.06 a	9.02 a	0.05 a
OTU5	*Phaeocollybia* (*Agaricales*, *Basidiomycota*), 100	0.10 a	0.10 a	6.96 a	0.80 a
OTU9	*Entoloma* (*Agaricales*, *Basidiomycota*), 100	7.23 a	0.27 a	0.04 a	0.05 a
OTU13	*Elaphomyces* (*Eurotiales*, *Ascomycota*), 100	8.38 a	0.04 a	0.06 a	0.07 a
OTU8	*Minimedusa* (*Cantharellales*, *Basidiomycota*), 52	0.04 a	0.05 a	0.09 a	6.28 a
OTU31	Unidentified Helotiales (*Helotiales*, *Ascomycota*), 100	2.19 a	6.02 a	0.03 a	0.02 a
OTU15	*Elaphomyces* (*Eurotiales*, *Ascomycota*), 99	5.93 a	0.04 a	0.06 a	0.06 a
OTU16	Unidentified Chytridiomycota (*Chytridiomycota*, Chytridiomycota), 10	2.33 a	3.83 a	0.02 a	0.03 a
OTU14	*Tylospora* (*Atheliales*, *Basidiomycota*), 100	0.85 a	0.59 a	3.05 a	1.17 a
OTU22	Unidentified Hygrophoraceae (*Agaricales*, *Basidiomycota*), 100	5.65 a	0.21 a	0.02 a	0.02 a
OTU11	*Elaphomyces* (*Eurotiales*, *Ascomycota*), 100	0.12 a	0.05 a	2.64 a	1.93 a
OTU28	Unidentified Agaricales (*Agaricales*, *Basidiomycota*), 98	4.45 a	0.27 a	0.03 a	0.03 a
OTU12	*Pannaria* (*Peltigerales*, *Ascomycota*), 51	0.02 a	0.03 a	2.89 a	1.53 a
OTU23	*Cortinarius* (*Agaricales*, *Basidiomycota*), 100	3.26 a	0.88 a	0.33 a	0.70 a
OTU58	*Geminibasidium* (*Geminibasidiales*, *Basidiomycota*), 100	1.84 b	2.89 b	0.21 a	0.21 a
OTU10	*Russula (Russulales, Basidiomycota*), 100	0.12 a	0.15 a	0.38 a	3.68 a
OTU39	*Russula (Russulales, Basidiomycota*), 100	4.16 a	0.15 a	0.03 a	0.03 a
OTU38	*Russula* (*Russulales*, *Basidiomycota*), 97	0.19 a	4.68 a	0.09 a	0.10 a
OTU20	*Mortierella* (*Mortierellales*, *Zygomycota*), 100	1.26 a	1.19 a	0.86 a	1.01 a
OTU34	*Russula* (*Russulales*, *Basidiomycota*), 100	2.91 a	0.85 a	0.09 a	0.07 a
OTU48	*Russula* (*Russulales*, *Basidiomycota*), 100	0.73 a	3.08 a	0.07 a	0.12 a
OTU51	*Russula* (*Russulales*, *Basidiomycota*), 100	1.99 a	1.91 a	0.04 a	0.05 a
OTU19	*Piloderma* (*Atheliales*, *Basidiomycota*), 100	0.05 a	0.04 a	1.33 a	2.00 a
OTU17	*Cortinarius* (*Agaricales*, *Basidiomycota*), 100	0.10 a	0.05 a	0.30 a	3.09 a
OTU32	*Sistotrema* (*Cantharellales*, *Basidiomycota*), 100	0.02 a	0.05 a	2.89 a	0.36 a
OTU21	*Terfezia* (*Pezizales*, *Ascomycota*), 100	2.30 a	1.40 a	0.02 a	0.02 a
OTU29	Unidentified Atheliaceae (*Atheliales*, *Basidiomycota*), 43	0.05 a	0.05 a	2.21 a	0.82 a
OTU187	Unidentified Pezizaceae (*Pezizales*, *Ascomycota*), 100	1.11 a	2.46 a	0.02 a	0.02 a
OTU61	*Mortierella* (*Mortierellales*, *Zygomycota*), 100	0.07 a	0.06 a	1.44 b	1.53 b
OTU44	*Humaria* (*Pezizales*, *Ascomycota*), 100	0.34 a	2.90 a	0.01 a	0.01 a
OTU25	*Pseudocraterellus* (*Cantharellales*, *Basidiomycota*), 97	0.16 a	3.57 a	0.01 a	0.02 a
OTU27	*Scleroderma* (*Boletales*, *Basidiomycota*), 100	0.01 a	3.61 a	0.01 a	0.01 a
OTU60	*Meliniomyces* (Incertae_sedis, *Ascomycota*), 100	0.03 a	0.05 a	0.64 a	2.25 a
OTU55	*Clavaria* (*Agaricales*, *Basidiomycota*), 59	3.19 a	0.11 a	0.02 a	0.02 a
OTU46	*Umbelopsis* (*Mucorales*, *Zygomycota*), 100	1.62 b	1.43 b	0.02 a	0.03 a
OTU79	*Tomentella* (*Thelephorales*, *Basidiomycota*), 96	0.97 a	2.35 a	0.02 a	0.04 a
OTU139	Unidentified Fungi (Unidentified Fungi, Unidentified Fungi), 16	0.98 a	2.10 a	0.01 a	0.01 a
OTU33	Unidentified Pezizomycetes (*Pezizomycetes*, *Ascomycota*), 100	0.04 a	0.04 a	1.26 a	1.25 a
OTU37	*Hydnum* (*Cantharellales*, *Basidiomycota*), 100	0.42 a	2.69 a	0.04 a	0.04 a
OTU30	*Tylospora* (*Atheliales*, *Basidiomycota*), 100	0.03 a	0.02 a	1.16 a	1.28 a
OTU66	*Hygrocybe* (*Agaricales*, *Basidiomycota*), 92	0.05 a	2.86 a	0.01 a	0.01 a
OTU45	*Tricholoma* (*Agaricales*, *Basidiomycota*), 100	1.21 a	1.54 a	0.03 a	0.02 a
OTU2642	*Amanita* (*Agaricales*, *Basidiomycota*), 100	0.11 a	2.67 a	0.01 a	0.01 a
OTU137	Unidentified Sporidiobolales (*Sporidiobolales*, *Basidiomycota*), 100	0.24 a	0.35 ab	0.76 ab	1.04 b
OTU97	*Hygrophorus* (*Agaricales*, *Basidiomycota*), 62	0.04 a	2.06 a	0.03 a	0.02 a
OTU47	*Cryptococcus* (*Filobasidiales*, *Basidiomycota*), 100	0.35 a	0.82 a	0.49 a	0.65 a
OTU42	Unidentified *Ascomycota* (*Ascomycota*, *Ascomycota*), 100	0.02 a	0.04 a	1.03 a	1.07 a
OTU54	*Pachyphloeus* (*Pezizales*, *Ascomycota*), 100	2.06 b	2.52 b	0.01 a	0.01 a
OTU199	Unidentified Pezizaceae (*Pezizales*, *Ascomycota*), 100	0.72 a	1.67 a	0.01 a	0.01 a
OTU41	Unidentified Agaricales (*Agaricales*, *Basidiomycota*), 100	1.80 a	0.61 a	0.01 a	0.01 a
OTU43	*Russula* (*Russulales*, *Basidiomycota*), 99	0.03 a	0.06 a	0.04 a	1.84 a
OTU64	Unidentified Chaetothyriales (*Chaetothyriales*, *Ascomycota*), 100	0.04 a	0.06 a	1.67 a	0.54 a
OTU68	*Tomentella* (*Thelephorales*, *Basidiomycota*), 98	0.20 a	1.81 a	0.02 a	0.02 a
OTU36	*Hydnum* (*Cantharellales*, *Basidiomycota*), 100	0.02 a	0.04 a	0.03 a	1.95 a
OTU83	*Tylospora* (*Atheliales*, *Basidiomycota*), 100	0.02 a	0.02 a	0.31 a	1.48 a
OTU26	*Pseudotomentella* (*Thelephorales*, *Basidiomycota*), 100	0.03 a	0.03 a	1.60 a	0.42 a
OTU74	*Tomentella* (*Thelephorales*, *Basidiomycota*), 100	1.55 a	0.60 a	0.01 a	0.02 a
OTU99	*Cortinarius* (*Agaricales*, *Basidiomycota*), 98	0.05 a	0.02 a	1.27 a	0.81 a
OTU75	*Rhizopogon* (*Boletales*, *Basidiomycota*), 99	1.90 a	0.17 a	0.04 a	0.02 a
OTU127	Unidentified *Ascomycota* (*Ascomycota*, *Ascomycota*), 100	0.03 a	0.02 a	0.96 b	0.83 b
OTU49	*Piloderma* (Atheliales, *Basidiomycota*), 100	0.94 a	1.01 a	0.07 a	0.04 a

OTU103	Unidentified Russulaceae (Russulales, *Basidiomycota*), 60	1.34 a	0.55 a	0.03 a	0.03 a
OTU105	Unidentified Fungi (Fungi), 100	1.02 a	0.87 a	0.01 a	0.02 a
OTU24	*Cortinarius* (*Agaricales*, *Basidiomycota*), 100	0.04 a	0.02 a	0.60 a	1.21 a
OTU107	*Boletus* (Boletales, *Basidiomycota*), 100	0.02 a	0.02 a	0.64 a	1.09 a
OTU57	Unidentified Fungi (Fungi), 100	1.40 a	0.45 a	0.01 a	0.03 a
OTU80	Unidentified Chaetothyriales (*Chaetothyriales*, *Ascomycota*), 100	0.38 a	1.71 a	0.02 a	0.03 a
OTU63	Unidentified Lecanoromycetes (*Lecanoromycetes*, *Ascomycota*), 100	0.00 a	0.00 a	0.57 ab	1.21 b
OTU18	*Clavulina* (*Cantharellales*, *Basidiomycota*), 100	0.02 a	0.04 a	1.14 a	0.74 a
OTU62	Unidentified Eurotiomycetes (*Eurotiomycetes*, *Ascomycota*), 100	0.99 a	1.07 a	0.01 a	0.00 a
OTU2494	*Cortinarius* (*Agaricales*, *Basidiomycota*), 97	1.39 a	0.12 a	0.10 a	0.12 a
OTU123	*Tretomyces* (*Atheliales*, *Basidiomycota*), 100	0.01 a	0.02 a	0.29 a	1.31 a
OTU88	*Cortinarius* (*Agaricales*, *Basidiomycota*), 100	0.31 a	0.02 a	0.36 a	0.91 a
OTU53	Unidentified *Ascomycota* (*Ascomycota*, *Ascomycota*), 100	0.16 a	1.60 a	0.05 a	0.09 a
OTU81	Unidentified Russulaceae (*Russulales*, *Basidiomycota*), 99	0.97 a	0.62 a	0.02 a	0.03 a
OTU52	*Lichtheimia* (*Mucorales*, *Zygomycota*), 90	0.62 a	1.15 a	0.01 a	0.01 a
OTU86	Unidentified Thelephoraceae (*Thelephorales*, *Basidiomycota*), 68	0.04 a	1.49 a	0.02 a	0.01 a
OTU143	*Umbelopsis* (*Mucorales*, *Zygomycota*), 100	0.78 a	0.79 a	0.13 a	0.11 a
OTU77	*Tomentella* (*Thelephorales*, *Basidiomycota*), 100	1.45 a	0.07 a	0.02 a	0.02 a
OTU116	Unidentified Agaricomycetes (*Agaricomycetes*, *Basidiomycota*), 82	1.46 a	0.04 a	0.02 a	0.02 a
OTU40	*Tomentella* (*Thelephorales*, *Basidiomycota*), 100	0.49 a	1.17 a	0.03 a	0.02 a
OTU59	Unidentified Eurotiomycetes (*Eurotiomycetes*, *Ascomycota*), 79	0.69 a	1.06 a	0.05 a	0.01 a
OTU35	Unidentified Helotiales (*Helotiales*, *Ascomycota*), 100	0.01 a	0.01 a	1.81 a	0.02 a
OTU126	*Tomentella* (*Thelephorales*, *Basidiomycota*), 96	0.11 a	1.71 a	0.02 a	0.02 a
OTU113	*Exophiala* (*Chaetothyriales*, *Ascomycota*), 100	0.43 a	1.20 a	0.02 a	0.01 a
OTU72	*Ophiostoma* (*Ophiostomatales*, *Ascomycota*), 56	0.00 a	0.00 a	0.13 a	1.23 a

Table S5. Taxonomic information of nodes categorized as generalists (module hubs and connectors) for the networks determined at the deciduous forest site M (545-570 m asl) and the coniferous forest site R (1,724-1,737 m) in spring and autumn using a 50% confidence threshold (ribosomal database project taxonomic classifier). Bacterial OTUs are identified by “B” after OTU number, while fungal OTUs are identified by “F”.

	Node ID	Type of Generalists	Domain	Phylum	Class	Order	Family
Site M							
	OTU236F	Module hubs	Fungi	Unclassified	Unclassified	Unclassified	Unclassified
	OTU446B	Module hubs	Bacteria	*Proteobacteria*	*Betaproteobacteria*	*Burkholderiales*	*Oxalobacteraceae*
	OTU221F	Module hubs	Fungi	*Ascomycota*	*Pezizomycetes*	*Pezizales*	*Pyronemataceae*
	OTU23B	Module hubs	Bacteria	*Acidobacteria*	Gp3	Gp3	Unclassified
	OTU238B	Module hubs	Bacteria	*Proteobacteria*	*Alphaproteobacteria*	*Rhizobiales*	Unclassified
	OTU4B	Module hubs	Bacteria	*Acidobacteria*	Gp1	Gp1	Unclassified
	OTU93B	Module hubs	Bacteria	*Planctomycetes*	*Planctomycetia*	*Planctomycetales*	*Planctomycetaceae*
	OTU219B	Module hubs	Bacteria	*Bacteroidetes*	*Sphingobacteriia*	*Sphingobacteriales*	*Chitinophagaceae*
	OTU94B	Module hubs	Bacteria	*Acidobacteria*	Gp2	Gp2	Unclassified
	OTU556B	Module hubs	Bacteria	*Bacteroidetes*	*Sphingobacteriia*	*Sphingobacteriales*	*Chitinophagaceae*
	OTU720F	Module hubs	Fungi	*Ascomycota*	*Sordariomycetes*	*Hypocreales*	*Nectriaceae*
	OTU507B	Module hubs	Bacteria	*Actinobacteria*	*Actinobacteria*	*Rubrobacteridae*	*Gaiellales*
	OTU1169B	Module hubs	Bacteria	*Acidobacteria*	Gp16	Gp16	Unclassified
	OTU534B	Module hubs	Bacteria	*Proteobacteria*	*Betaproteobacteria*	Unclassified	Unclassified
	OTU100B	Module hubs	Bacteria	*Proteobacteria*	*Betaproteobacteria*	*Burkholderiales*	*Incertae sedis*
	OTU461B	Module hubs	Bacteria	*Proteobacteria*	*Alphaproteobacteria*	*Caulobacterales*	*Hyphomonadaceae*
	OTU54B	Module hubs	Bacteria	*Proteobacteria*	*Gammaproteobacteria*	*Xanthomonadales*	*Xanthomonadaceae*
	OTU139F	Module hubs	Fungi	Unclassified	Unclassified	Unclassified	Unclassified
	OTU630B	Module hubs	Bacteria	*Proteobacteria*	*Gammaproteobacteria*	Candidatus *Carsonella*	Unclassified
	OTU65B	Connectors	Bacteria	*Bacteroidetes*	*Sphingobacteriia*	*Sphingobacteriales*	*Chitinophagaceae*
	OTU1616B	Connectors	Bacteria	*Actinobacteria*	*Actinobacteria*	*Rubrobacteridae*	*Solirubrobacterales*
	OTU241B	Connectors	Bacteria	*Proteobacteria*	*Alphaproteobacteria*	*Rhizobiales*	*Phyllobacteriaceae*
	OTU538B	Connectors	Bacteria	*Proteobacteria*	*Gammaproteobacteria*	*Xanthomonadales*	*Xanthomonadaceae*
	OTU953B	Connectors	Bacteria	*Proteobacteria*	*Deltaproteobacteria*	*Myxococcales*	*Nannocystineae*
	OTU3367B	Connectors	Bacteria	*Proteobacteria*	*Deltaproteobacteria*	*Bdellovibrionales*	*Bdellovibrionaceae*
	OTU636B	Connectors	Bacteria	*Bacteroidetes*	*Sphingobacteriia*	*Sphingobacteriales*	*Chitinophagaceae*
	OTU778B	Connectors	Bacteria	*Proteobacteria*	*Alphaproteobacteria*	*Rhizobiales*	*Hyphomicrobiaceae*
	OTU55B	Connectors	Bacteria	WPS-2	*WPS-2*	Unclassified	Unclassified
	OTU651B	Connectors	Bacteria	Unclassified	Unclassified	Unclassified	Unclassified
	OTU165B	Connectors	Bacteria	*Actinobacteria*	*Actinobacteria*	*Actinobacteridae*	*Actinomycetales*
	OTU1032B	Connectors	Bacteria	*Bacteroidetes*	*Sphingobacteriia*	*Sphingobacteriales*	*Chitinophagaceae*
	OTU609B	Connectors	Bacteria	*Bacteroidetes*	*Sphingobacteriia*	*Sphingobacteriales*	*Chitinophagaceae*

	OTU35B	Connectors	Bacteria	*Acidobacteria*	Gp2	Gp2	Unclassified
	OTU119B	Connectors	Bacteria	*Proteobacteria*	*Alphaproteobacteria*	*Rhodospirillales*	*Rhodospirillaceae*
	OTU467B	Connectors	Bacteria	*Acidobacteria*	Gp3	Gp3	*Unclassified*
	OTU222F	Connectors	Fungi	*Ascomycota*	*Leotiomycetes*	*Helotiales*	*Unidentified Helotiales*
	OTU246B	Connectors	Bacteria	*Bacteroidetes*	*Sphingobacteriia*	*Sphingobacteriales*	*Sphingobacteriaceae*
	OTU33F	Connectors	Fungi	*Ascomycota*	*Pezizomycetes*	Unclassified	Unclassified
	OTU1333B	Connectors	Bacteria	*Firmicutes*	Unclassified	Unclassified	Unclassified
Site R							
	OTU470F	Module hubs	Fungi	*Basidiomycota*	*Gammaproteobacteria*	Candidatus *Carsonella*	Unclassified
	OTU630B	Module hubs	Bacteria	*Proteobacteria*	Unclassified	Unclassified	Unclassified
	OTU445F	Module hubs	Fungi	Unclassified	*Agaricomycetes*	Unclassified	Unclassified
	OTU293F	Module hubs	Fungi	*Basidiomycota*	*Eurotiomycetes*	*Eurotiales*	Unclassified
	OTU1426F	Module hubs	Fungi	*Ascomycota*	Unclassified	*Unclassified*	*Elaphomycetaceae*
	OTU438B	Module hubs	Bacteria	Unclassified	*Actinobacteria*	*Acidimicrobidae*	Unclassified
	OTU437B	Connectors	Bacteria	*Actinobacteria*	*Gammaproteobacteria*	Candidatus *Carsonella*	*Acidimicrobiales*

Table S6. Normalized signal intensities of the detected genes using Geochip 5.0 at the deciduous forest site M (545-570 m asl) and the coniferous forest site R (1,724-1,737 m) in spring and autumn and grouped according to their funcional categories. For each functional category, values followed by different letters are significantly different (p≤0.05) according to Tukey's HSD test. Total relative percentage of each gene category considering the 12 samples analyzed is also provided.

	Site M		Site R		Total relative percentage
**Functional group**	Spring	Autumn	Spring	Autumn	
Carbon cycling	221.49 a	229.90 bc	228.62 b	233.12 c	41.88
Organic remediation	124.31 a	127.89 b	125.91 ab	129.05 b	23.26
Nitrogen cycling	68.75 a	70.81 b	70.44 b	71.41 b	12.91
Sulfur cycling	42.49 a	43.80 b	43.88 b	44.97 c	8.03
Metal homeostasis	38.91 a	39.90 ab	39.90 ab	40.90 b	7.32
Phosphorus cycling	23.12 a	24.40 b	24.07 b	24.70 b	4.42
Virulence	8.95 a	9.15 a	9.02 a	9.16 a	1.66
Other functions	2.01 a	2.04 a	1.87 a	1.87 a	0.36
Secondary metabolism	0.89 a	0.93 a	0.95 a	0.97 a	0.17

Table S7. Normalized signal intensities of genes related to C degradation detected at the deciduous forest site M (545-570 m asl) and the coniferous forest site R (1,724-1,737 m) in spring and autumn. The targeted substrates were arranged in order from labile to recalcitrant C. For each gene, values followed by different letters are significantly different (p≤0.05) according to Tukey's HSD test.

		Site M		Site R	
C source	Genes	Spring	Autumn	Spring	Autumn
Simple sugars	Glucose oxidase	0.27 a	0.37 b	0.35 b	0.38 b
Simple sugars	Invertase	0.10 ab	0.07 a	0.10 ab	0.13 b
Starch	*amyA*	50.74 a	52.99 b	52.77 b	54.00 c
Starch	Glucoamylase	2.23 a	2.29 a	2.27 a	2.36 a
Starch	*cda*	1.88 a	1.91 a	2.05 a	1.93 a
Starch	*pulA*	1.36 a	1.37 a	1.56 b	1.61 b
Starch	*apu*	0.05 a	0.05 a	0.07 a	0.05 a
Starch	*amyS*	0.03 a	0.04 b	0.04 b	0.07 c
Hemicellulose	*ara*	7.44 a	8.00 b	7.90 b	8.00 b
Hemicellulose	Xylanase	6.44 a	6.72 b	6.53 ab	6.59 c
Hemicellulose	*xylA*	4.64 a	4.69 a	4.89 b	5.00 b
Hemicellulose	Mannanase	3.20 a	3.50 c	3.19 a	3.33 b
Cellulose	Cellobiase	5.46 a	5.68 a	6.12 b	6.15 b
Cellulose	Endoglucanase	3.44 b	3.45 b	3.37 a	3.48 b
Cellulose	Exoglucanase	1.51 a	1.44 a	1.69 b	1.83 c
Chitin	Chitinase	10.71 a	11.61 b	10.89 a	11.85 b
Chitin	Acetylglucosaminidase	7.83 a	8.06 a	7.88 a	7.79 a
Chitin	Endochitinase	3.18 a	3.26 a	3.25 a	3.56 b
Chitin	Exochitinase	0.39 a	0.48 b	0.37 a	0.36 a
Chitin	Chitin deacetylase	0.31 a	0.27 a	0.27 a	0.27 a
Pectin	*pme CDeg*	3.62 a	3.63 a	3.60 a	3.94 b
Pectin	*rgaE*	3.15 a	3.40 b	3.33 b	3.09 a
Pectin	Pectinase	2.65 a	2.62 a	2.67 a	2.84 b
Pectin	*rgl*	2.47 a	2.56 ab	2.71 b	2.59 ab
Pectin	*pec_Cdeg*	1.17 a	1.27 b	1.23 b	1.28 b
Pectin	Exopolygalacturonase	0.66 a	0.75 b	0.71 ab	0.75 b
Pectin	*rgh*	0.71 a	0.66 a	0.68 a	0.70 a
Pectin	Endopolygalacturonase	0.65 c	0.56 b	0.48 a	0.55 b
Pectin	*pel_Cdeg*	0.25 a	0.30 b	0.30 b	0.30 b
Pectin	Pectin lyase	0.11 a	0.16 b	0.17 b	0.22 c
Aromatics	*vanA*	3.44 a	3.45 a	3.42 a	3.52 a
Aromatics	*limEH*	1.61 a	1.86 b	1.63 a	1.70 a
Aromatics	*cdh*	1.47 a	1.55 a	1.39 a	1.49 a
Aromatics	*vdh*	1.10 a	1.14 a	1.12 a	1.16 a
Aromatics	*camDCAB*	0.26 a	0.26 a	0.28 a	0.29 a
Aromatics	*lmo*	0.12 a	0.12 a	0.12 a	0.15 b
Lignin	Phenol oxidase	4.98 a	5.21 a	5.14 a	5.58 b
Lignin	*mnp*	0.85 a	0.80 a	0.84 a	0.94 b
Lignin	*glx*	0.79 b	0.84 b	0.80 b	0.68 a
Lignin	Ligninase	0.36 a	0.39 a	0.34 a	0.37 a

Table S8. Normalized signal intensities of key genes involved in sulfur and phosphorus cycling as well as in organic remediation detected using Geochip 5.0 and found at the deciduous forest site M (545-570 m asl) and the coniferous forest site R (1,724-1,737 m) in spring and autumn. For each functional gene category, values followed by different letters are significantly different ($p \leq 0.05$) according to Tukey's HSD test.

		Site M		Site R	
Functional group	Genes	Spring	Autumn	Spring	Autumn
**Sulfur cycling**					
Sulfite reduction	*dsrA, dsrB, sir*	19.19 a	19.53 a	19.46 a	20.30 b
Sulfur oxidation	*soxA, soxB, soxC, soxY,*	6.55 a	6.91 bc	6.67 ab	6.96 c
Sulfite reduction	*cysJ*	5.86 a	5.88 a	6.12 b	6.13 b
Adenylylsulfate reductase	*APS_AprA, APS_aprB*	3.50 ab	3.53 b	3.56 b	3.34 a
Sulfide Oxidation	*sqr, fccAB*	2.70 a	2.92 b	3.11 c	3.07 bc
DMSP degradation	*dmdA*	1.06 a	1.08 ab	1.09 ab	1.17 b
**Phosphorus cycling**					
Polyphosphate degradation	*ppx*	15.48 a	16.07 b	15.88 ab	16.26 b
Polyphosphate synthesis	*ppk*	5.11 a	5.59 b	5.48 b	5.78 c
Phytic acid hydrolysis	*phytase*	2.54 a	2.74 a	2.70 a	2.66 a
**Organic remediation**					
Aromatics[1]	*amiE, arylest, badH, bco, benD, bph,bphB, bphC, bphD, bphF1, catB, catechol, cmcI, mdlA, mdlB, mdlC, mhpA, mhpB, nagG, nahF, nhh, nitrilase, one_ring_23diox,pcaG, pobA, tfdA, xyl,*	81.07 a	84.12 b	82.17 ab	84.17 b
Herbicides related compound	*phn, tcpA, trzA, trzE, trzN*	13.50 bc	13.10 a	13.32 ab	13.76 c
Other Hydrocarbons	*alkB, assA, chnA, chnB, chnC, chnD, chnE, cpnA, cpnB, cpnC, cpnE, xamo*	10.39 a	10.87 b	10.56 ab	10.86 b
Chlorinated solvents	*cmuA, dehH, dehH109, exaA,*	7.86 a	8.24 a	8.12 a	7.93 a
Pesticides related compound	*adpB, linB, linC,*	5.95 a	5.98 a	5.96 a	6.31 b
Halogenated compounds	*Dyp*	0.17 a	0.24 b	0.31 b	0.25 c

[1] Only most abundant genes have been described

Table S9. Mantel test results correlating bacterial and fungal abundance, diversity (Shannon index) and community structures with soil physicochemical properties and soil temperature measurements. R^2 values in bold indicate statistical significance ($p \leq 0.05$). Significance levels are shown at *$p \leq 0.05$, **$p \leq 0.01$, ***$p \leq 0.001$

	Bacterial abundance	Fungal abundance	Bacterial diversity	Fungal diversity	Bacterial community structure	Fungal community structure
**Soil physicochemical properties**						
pH	**0.7952*****	**0.6523*****	**0.7986*****	**0.6895*****	**0.8472*****	**0.6354*****
EC[1]	**0.6584*****	**0.5935*****	**0.6514*****	**0.5981*****	**0.5991*****	**0.4529*****
SOM[2]	**0.6125*****	**0.5825*****	**0.6952*****	**0.5744*****	**0.6938*****	**0.4828*****
TOC[3]	**0.6215*****	**0.5912*****	**0.7085*****	**0.5825*****	**0.6931*****	**0.4931*****
N	**0.4925*****	**0.4125*****	**0.4615*****	**0.3915*****	**0.5928*****	**0.4333*****
NH_4^+-N	0.1112	0.0916	0.0725	0.0456	0.1202	0.0650
NO_3^--N	**0.2951*****	**0.2851*****	**0.2524*****	**0.2125*****	**0.3096*****	**0.2641*****
C/N	**0.1921*****	**0.2085*****	**0.1825*****	**0.1760*****	**0.2100****	**0.2021****
P	0.1125	0.0852	0.0984	0.0954	0.1098	0.0695
K	**0.4925*****	**0.4621*****	**0.4651*****	**0.3952*****	**0.5343*****	**0.3931*****
Mg	0.0001	-0.0052	-0.0051	0.0089	-0.0790	-0.0179
**Soil temperature**						
Mean soil temperature	**0.2351****	**0.3152*****	**0.326****	**0.2699****	**0.2145****	**0.1925****
Maximum soil temperature	**0.3125****	**0.2189****	**0.2142****	**0.2587****	**0.3102****	**0.2456****
Minium soil temperature	**0.2156****	**0.2546****	**0.2954****	**0.2698****	**0.3021****	**0.2369****

[1] EC, electrical conductivity
[2] SOM, soil organic matter
[3] TOC, total organic carbon

Fig. S1. Relative abundance of the different bacterial (a) and fungal (b) classes found at the deciduous forest site M (545-570 m asl) and the coniferous forest site R (1,724-1,737 m) in spring and autumn.

a)

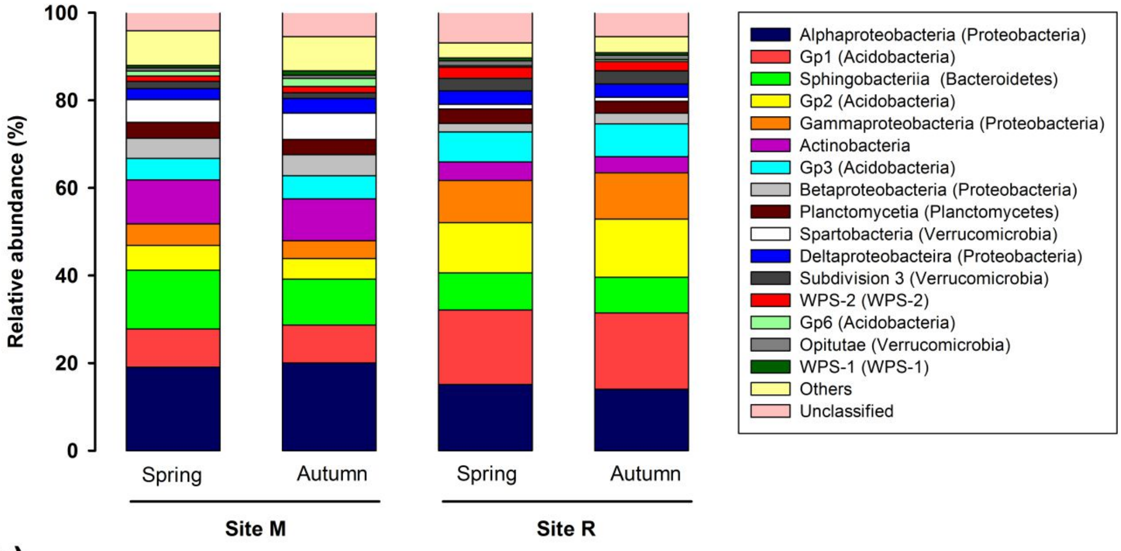

b)

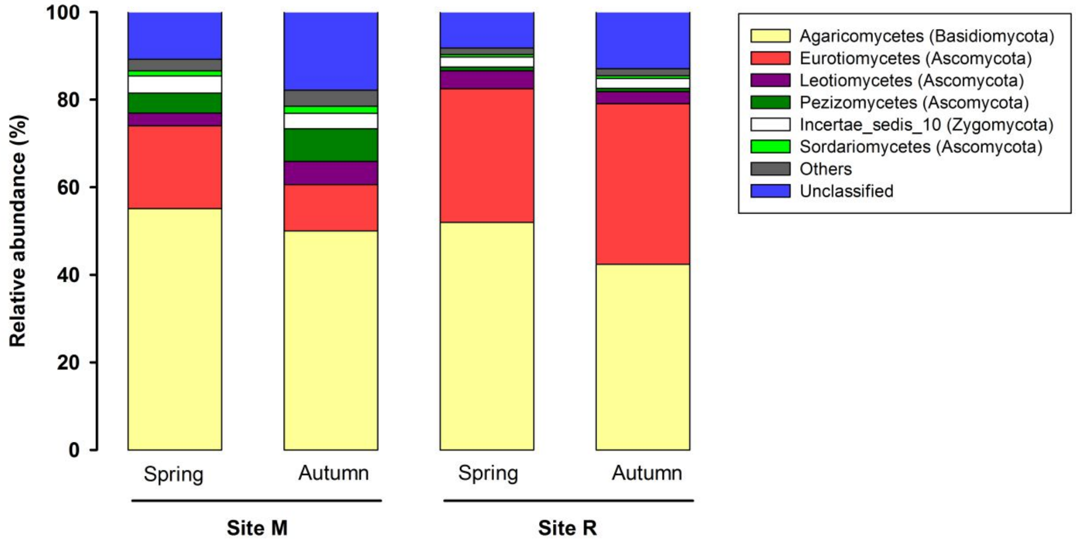

Fig. S2. Zi-Pi plot for the deciduous forest site M (545-570 m asl) (a) and the coniferous forest site R (1,724-1,737 m) (b) showing the distribution of OTUs based on their topological roles. Each symbol represents a bacterial or fungal OTU at each forest site. The threshold values of Zi and Pi were 2.5 and 0.62, respectively. Generalists OTUs (either module hubs or connectors) were identified.

a)

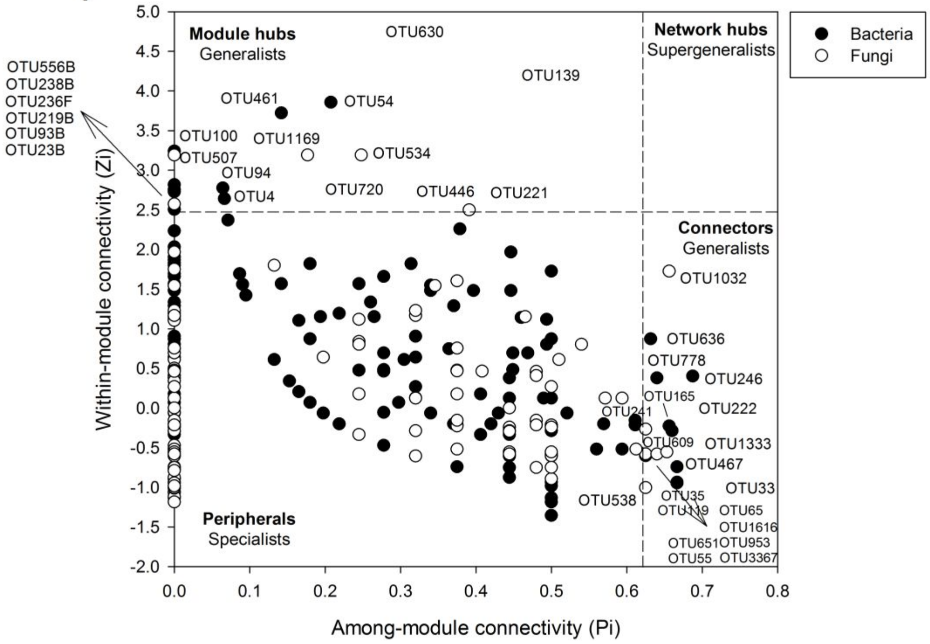

b)

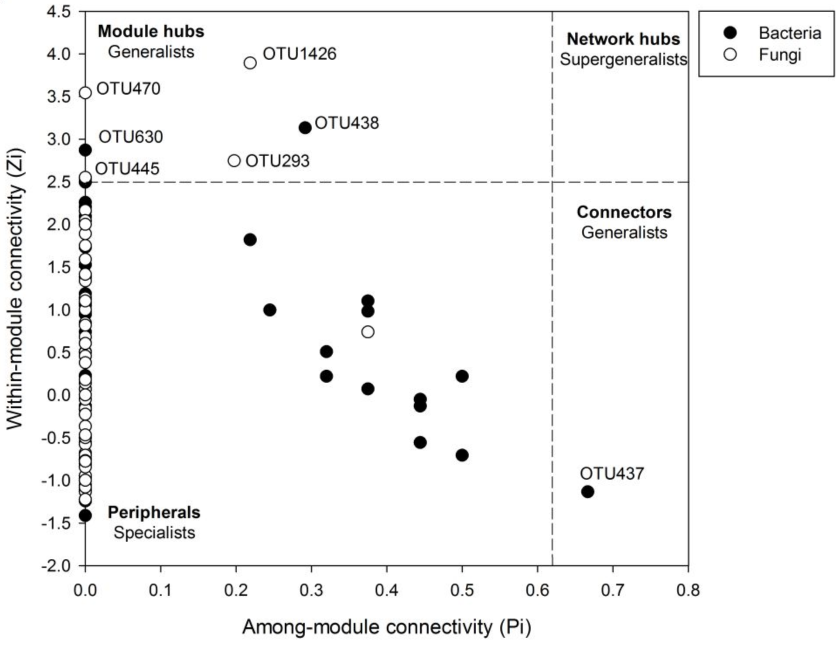

Supplement: Supplementary file 1 — Supplementary Information [file 41598_2017_2363_MOESM1_ESM.pdf]
